# Effectiveness of non-pharmacological therapies in complementary and alternative medicine on improving fatigue levels, oxidative stress, inflammation, and endocrine levels in animal models of chronic fatigue-like conditions: a systematic review and network meta-analysis

**DOI:** 10.3389/fphys.2026.1807587

**Published:** 2026-05-07

**Authors:** Shumeng Ren, Jingyuan Wei, Shan Zhao, Linghua Li, Jingshi Zhang, Chaoqun Yan, Jun Wang

**Affiliations:** Department of Acupuncture and Moxibustion, Dongzhimen Hospital of Beijing University of Chinese Medicine, Beijing, China

**Keywords:** animals, complementary and alternative medicine, fatigue, network meta-analysis, non-pharmacological therapy

## Abstract

**Objective:**

To systematically evaluate the effects of various complementary and alternative medicine (CAM) non-pharmacological therapies on fatigue levels, oxidative stress, inflammation, and endocrine indicators in chronic fatigue-like conditions animal models, and to rank the efficacy of these interventions.

**Methods:**

A computerized search was conducted across databases including PubMed, Cochrane Library, Embase, Web of Science, CNKI, Wanfang, VIP, and CBMdisc, to collect animal experiments on fatigue-like conditions treated with CAM non-pharmacological therapies published from the establishment of the databases to January 14, 2026. Two researchers independently screened the literature, extracted data, and assessed risk of bias. Stata 16.0 software was used for network meta-analysis, and SYRCLE’s risk of bias tool was employed to evaluate the quality of the included studies.

**Results:**

A total of 77 studies involving nine types of CAM non-pharmacological therapies were included. In terms of improving fatigue levels, compared to the control group, electroacupuncture (MD = 347.00 s, 95% CI [144.70, 549.29]) and moxibustion (MD = 311.28 s, 95% CI [146.36, 476.20]) significantly prolonged exhaustive swimming time. In terms of improving oxidative stress damage, fire acupuncture (MD = -13.15 nmol/ml, 95% CI [-18.06, -8.24]), manual acupuncture (MD = -3.85 nmol/ml, 95% CI [-5.12, -2.58]), needle-pricking (MD = -11.43 nmol/ml, 95% CI [-16.54, -6.32]), and moxibustion (MD = -479.16 nmol/ml, 95% CI [-567.05, -391.27]) significantly reduced MDA levels. In terms of improving inflammatory damage, Tuina (MD = -552.03 pg/ml, 95% CI [-700.81, -403.25]) and electroacupuncture (MD = -156.59 pg/ml, 95% CI [-259.85, -53.34]) can significantly reduce the level of IL-1β. In terms of regulating endocrine, electroacupuncture (MD = -9.91 pg/ml, 95% CI [-14.77, -5.05]) and Tuina (MD = -16.96 pg/ml, 95% CI [-25.37, -8.55]) can significantly reduce the level of CRH.

**Conclusion:**

Non-pharmacological therapies in complementary and alternative medicine have great potential in improving fatigue-related phenotypes, oxidative stress damage, inflammatory damage, and regulating endocrine levels in animal models of fatigue-like conditions. Future research should focus on developing animal models that better replicate the pathogenesis and core characteristics of CFS, and then extend them to randomized controlled trials involving CFS patients to verify the transformation potential.

**Systematic review registration:**

https://www.crd.york.ac.uk/prospero/, identifier CRD420251177070.

## 1 Introduction

Chronic fatigue syndrome (CFS) is a complex, multisystem chronic disease characterized by debilitating fatigue that worsens following physical exertion. It is commonly accompanied by a constellation of symptoms including post-exertional malaise, non-restorative sleep, cognitive impairment, orthostatic intolerance, widespread pain, and sensory hypersensitivity. These manifestations significantly impair patients’ occupational functioning, social engagement, and activities of daily living, thereby imposing a substantial public health burden (National Institute for Health and Care Excellence, 2021). Epidemiological data indicate that approximately 25% of adults in the United States report experiencing chronic fatigue (Galland-Decker et al., 2019). In England’s primary care population, the minimum prevalence of CFS is 0.2%, with an annual incidence of 0.015%; notably, incidence rates are higher among women and in the London region (Nacul et al., 2011). Similarly, a Chinese study of 301 patients with “fatigue of unknown origin” revealed that fatigue was the primary reason for seeking medical care in over half of the cases (Liu et al., 2020).

The pathophysiological mechanisms underlying CFS remain incompletely elucidated. Current research has primarily focused on hypothalamic-pituitary-adrenal (HPA) axis dysfunction, immune-inflammatory activation, and metabolic abnormalities (Cleare, 2004; Hornig et al., 2015). In recent years, energy metabolism disturbances—particularly impaired pyruvate dehydrogenase function and mitochondrial dysfunction—have emerged as key contributors to post-exertional malaise and other CFS symptoms (Fluge et al., 2016). Presently, clinical management relies predominantly on cognitive behavioral therapy, graded exercise therapy, and limited pharmacological interventions (e.g., analgesics and antidepressants) (Vink and Vink-Niese, 2018; Larun et al., 2019). Consequently, the exploration of safe, effective, and multi-target alternative therapeutic approaches represents a critical research priority.

Complementary and alternative medicine (CAM) non-pharmacological therapies have demonstrated considerable potential in CFS management. Modalities such as electroacupuncture, manual acupuncture, and moxibustion appear to exert systemic regulatory effects through multiple pathways, including immunomodulation, neuroinflammation reduction, energy metabolism enhancement, and HPA axis regulation (Zhang et al., 2019; Yang et al., 2024; Zheng et al., 2024). Owing to their favorable safety profiles and minimal adverse effects, these interventions are increasingly recognized as promising adjunctive treatment options for CFS. Preclinical studies have provided preliminary evidence of their efficacy in ameliorating fatigue-related behaviors and biochemical markers in animal models. However, existing investigations are generally limited by small sample sizes and heterogeneous stimulation protocols, rendering direct comparisons problematic and precluding systematic evaluation of the relative efficacy of different interventions. Furthermore, it is important to note that current animal models, primarily induced by forced swimming, sleep deprivation, or dietary restriction, simulate fatigue-like phenotypes rather than fully replicating the complex, multi-system pathophysiology of human CFS.

Network meta-analysis (NMA) enables the integration of direct and indirect comparative evidence, facilitating the assessment and ranking of multiple interventions within a unified analytical framework (Cipriani et al., 2013). This methodology has proven valuable in evaluating CAM treatments for various diseases, yet it has not been applied to the comprehensive assessment of CAM non-pharmacological interventions in animal models of fatigue-like conditions. Therefore, this study proposes to employ NMA for the first time to systematically evaluate the effects of various CAM non-pharmacological interventions on key outcomes—including fatigue behavior, oxidative stress, inflammatory markers, and endocrine parameters—in these animal models. Additionally, this analysis will generate efficacy rankings for the interventions examined, with the aim of providing high-quality evidence and methodological guidance for optimizing future basic research designs and ultimately facilitating clinical translation for CFS patients.

## 2 Methods

This systematic review and network meta-analysis protocol has been registered with the International Prospective Register of Systematic Reviews (PROSPERO; registration number: CRD420251177070) and was reported in accordance with the Preferred Reporting Items for Systematic Reviews and Meta-Analyses extension for Network Meta-Analyses (PRISMA-NMA) guidelines (Hutton et al., 2015).

### 2.1 Search strategy

Two independent reviewers (RenSM and WeiJY) conducted comprehensive systematic searches across eight electronic databases: PubMed, Cochrane Library, Embase, Web of Science, CNKI, Wanfang, VIP, and CBMdisc. The search timeframe extended from database inception to January 14, 2026. The complete search strategy is provided in **Supplementary File 1**. The search strategy was initially developed for PubMed and subsequently adapted to the specific characteristics of each database. Following independent screening, any disagreements between reviewers were resolved through consensus discussion or arbitration by a third reviewer (WangJ).

### 2.2 Eligibility criteria

Study selection was guided by the PICOS framework, with detailed criteria presented in **Table 1**.

**Table 1:** PICOS criteria.

Criteria	Explanation
Participants	Animal models of fatigue-like conditions
Intervention	Non-pharmacological therapies in complementary and alternative medicine
Comparison	Conventional therapy, placebo, or no intervention
Outcomes	Exhaustion time in forced swim test, immobility time in tail suspension test, escape latency in morris water maze, vertical score in open field test, horizontal score in open field test, body weight, SOD, MDA, IL-1β, IL-6, TNF-α, IFN-γ, CRH, ACTH, CORT

SOD, superoxide dismutase activity; MDA, malondialdehyde concentration; IL-1β, interleukin-1β level; IL-6, interleukin-6 level; TNF-α, tumor necrosis factor-α level; IFN-γ, interferon-γ level; CRH, corticotropin-releasing hormone level; ACTH, adrenocorticotropic hormone level; CORT, cortisol level.

#### 2.2.1 Inclusion criteria

1. Study subjects: Experimental animals with successfully induced fatigue-like conditions using established modeling methods (e.g. forced swimming, sleep deprivation, dietary restriction, or combined stressors), without restrictions on species, sex, age, or induction method;
2. Interventions: Complementary and alternative medicine (CAM) non-pharmacological therapies, including manual acupuncture, electroacupuncture, fire acupuncture, moxibustion, warm needling therapy, tuina (Chinese therapeutic massage), guasha (scraping therapy), thread-embedding therapy, and needle-pricking therapy;
3. Experimental design: Studies must include both a fatigue-like condition model group and a treatment group;
4. Outcome measures: Exhaustion time in forced swim test, immobility time in tail suspension test, escape latency in morris water maze, vertical score in open field test, horizontal score in open field test, body weight, superoxide dismutase (SOD) activity, malondialdehyde (MDA) concentration, interleukin-1β (IL-1β) level, interleukin-6 (IL-6) level, tumor necrosis factor-α (TNF-α) level, interferon-γ (IFN-γ) level, corticotropin-releasing hormone (CRH) level, adrenocorticotropic hormone (ACTH) level, and cortisol (CORT) level.

#### 2.2.2 Exclusion criteria

1. Duplicate publications or redundant data;
2. Clinical trials or human studies;
3. Dissertations, conference abstracts, letters, case reports, and studies with inaccessible full texts;
4. Animal models with comorbid conditions;
5. *In vitro* experiments, ex vivo studies, and computer simulation studies.

### 2.3 Data extraction and quality assessment

Data extraction was performed independently by two reviewers (ZhaoS and LiLH). Any disagreements were resolved through consensus discussion or arbitration by a third reviewer (WangJ).

The following data were extracted: (1) author information; (2) publication year; (3) country/region; (4) animal model details (species, age, sex, body weight, sample size, modeling method); (5) intervention characteristics (intervention type, frequency, acupoint selection protocol); (6) sample type; (7) implementation of blinding and randomization; (8) outcome measures. For studies with multiple reports or different follow-up periods from the same animal experiment, measurements obtained immediately post-intervention were selected as the experimental outcomes. Baseline and immediate post-intervention data were prioritized, with mean change and standard deviation from baseline to end of intervention extracted as the primary data for analysis. When standard deviations were not reported, they were calculated from standard errors, 95% confidence intervals, ranges, or interquartile ranges using established formulas (detailed in **Supplementary File 2**). All extracted data were recorded in a pre-designed structured data collection form. In this study, the control group was defined as animals that did not receive the experimental intervention, comprising both blank control groups and Western medicine treatment groups. Specific intervention definitions and control group classifications are detailed in **Supplementary Table 3**.

Quality assessment was conducted independently by two reviewers (RenSM and WeiJY), with discrepancies resolved by a third reviewer (YanCQ). Risk of bias in animal studies was evaluated using the SYRCLE (Systematic Review Centre for Laboratory Animal Experimentation) risk of bias tool (Hooijmans et al., 2014). Assessment domains included selection bias, performance bias, detection bias, attrition bias, reporting bias, and other sources of bias.

### 2.4 Data analysis

Statistical analyses were performed using Stata 16.0 software (StataCorp LLC, College Station, TX, USA). Network meta-analysis was conducted using the mvmeta package within a frequentist framework to evaluate the relative efficacy of various complementary and alternative medicine (CAM) non-pharmacological therapies in animal models of fatigue-like conditions. Continuous data were analyzed using mean ± standard deviation as the effect measure. For studies with multiple intervention groups, these were divided into multiple independent pairwise comparisons to preserve the integrity of the network structure.

Consistency between direct and indirect comparisons was assessed using the node-splitting method. A P-value > 0.05 indicated acceptable consistency, and the consistency model was employed for effect size pooling; if significant inconsistency was detected (P ≤ 0.05), the inconsistency model was applied and results were reported accordingly. Statistical heterogeneity among studies was quantified using the *I²* statistic, with *I²* > 50% considered indicative of substantial heterogeneity. To ensure conservative estimates, a random-effects model was applied for all analyses regardless of heterogeneity magnitude. Finally, the relative ranking probabilities of interventions were determined by calculating the surface under the cumulative ranking curve (SUCRA), with higher SUCRA values indicating greater probability of being the optimal intervention.

## 3 Results

### 3.1 Literature search results

**Figure 1:**
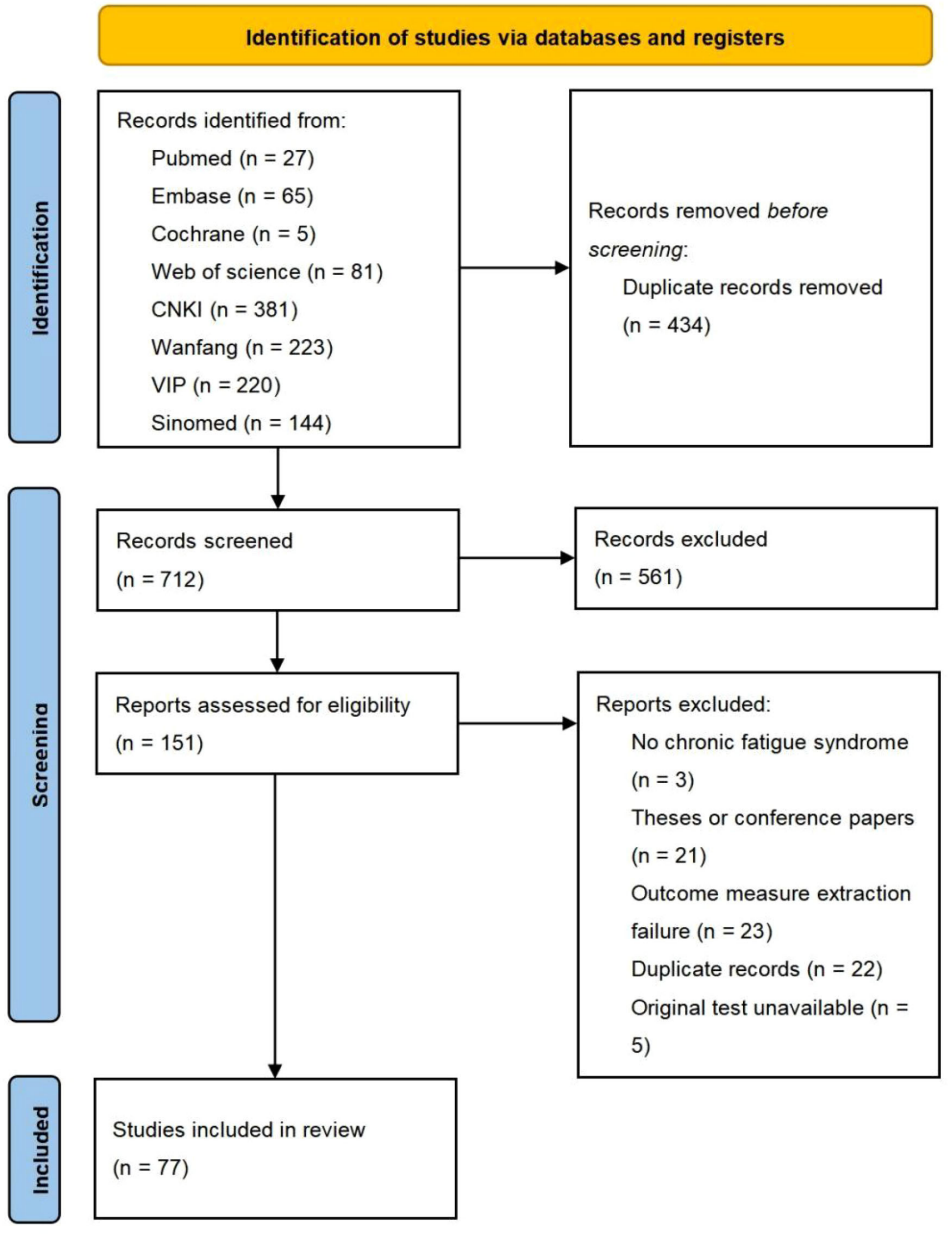
PRISMA diagram.

**Figure 1** presents the PRISMA flow diagram detailing the literature screening process. A total of 1,146 potentially relevant records were identified through systematic searching. Following duplicate removal and title/abstract screening to exclude obviously ineligible studies, the full texts of remaining articles were assessed for eligibility. Ultimately, 77 studies (Tang et al., 2007; Qu et al., 2010; Yao and Fang, 2011; Cheng et al., 2012; Cheng et al., 2013; Qian et al., 2018; Wang et al., 2018; Liu et al., 2021; Liu and Lei, 2022; An et al., 2024; Liu and Lei, 2024; Zhai et al., 2025a; Zhai et al., 2025b) met the inclusion criteria and were included in the network meta-analysis. Of these, 75 were published in Chinese and 2 (Meng et al., 2003a; Qian et al., 2019) in English, with publication years spanning from 2003 to 2025. All studies were animal experiments investigating nine types of complementary and alternative medicine (CAM) non-pharmacological therapies: manual acupuncture, electroacupuncture, fire acupuncture, moxibustion, warm needling therapy, tuina, guasha, thread-embedding therapy, and needle-pricking therapy. The detailed characteristics of included studies are provided in **Supplementary Table 4**.

### 3.2 Quality assessment

The methodological quality of 77 included animal experiments was systematically evaluated using the SYRCLE risk of bias tool. The detailed results are as follows:

(1) Selection bias. For sequence generation, 50% of studies (n = 39) (Meng et al., 2003b; Lv and Lv, 2009a; Lv and Lv, 2009b; Zou et al., 2010; Yao and Fang, 2011; Zeng et al., 2011; Cheng et al., 2012; Lv et al., 2012; Cheng et al., 2013; Sun et al., 2014; Zhai et al., 2016; Qian et al., 2018; Li et al., 2019a; Li et al., 2019b; Li et al., 2025) were rated as low risk due to explicit use of randomization methods (e.g., random number tables); the remaining studies merely mentioned “randomization” without specifying methods and were rated as unclear risk. For baseline characteristics, 63% (n = 49) (Wang et al., 2004; Wu et al., 2006; Lv and Lv, 2009a; Lv and Lv, 2009b; Chen et al., 2010; Qu et al., 2010; Zhu, 2010; Zou et al., 2010; Yao and Fang, 2011; Cheng et al., 2012; Luo and Luo, 2012; Lv et al., 2012; Cheng et al., 2013; Zeng et al., 2013a; Zeng et al., 2013b; Sun et al., 2014; Yang et al., 2014; Zhao and Jiang, 2014; Yang et al., 2016; Zhai et al., 2016; Hua et al., 2017b; Hua et al., 2017a; Liu and Jin, 2018; Qian et al., 2018; Yang et al., 2018; Zhou et al., 2018a; Zhou et al., 2018b; Li et al., 2019b; Qian et al., 2019; Si et al., 2020; Yi et al., 2020; Liu et al., 2021; Pan et al., 2021; Shui et al., 2021a; Shui et al., 2021b; Han et al., 2023; Su et al., 2024; Li et al., 2025; Zhai et al., 2025a; Zhai et al., 2025b) reported between-group comparability or statistical adjustments and were rated as low risk; 37% without relevant information were rated as unclear risk. No study described allocation concealment, resulting in unclear risk ratings for all.

(2) Performance bias. For random housing, 13% (n = 10) (Chen et al., 2009a; Luo and Luo, 2012; Mao et al., 2013; Zhao and Jiang, 2014; Hua et al., 2017a; Zhou et al., 2018a; Qian et al., 2019; Shui et al., 2021a; Shui et al., 2021b; Han et al., 2023) used non-random cage allocation, introducing potential systematic errors and warranting high risk ratings; 87% without housing information were rated as unclear risk. For blinding of participants and personnel, 50% (n = 38) (Meng et al., 2003a; Meng et al., 2003b; Wu et al., 2006; Chen et al., 2009a; Chen et al., 2009b; Chen et al., 2010; Qu et al., 2010; Yao and Fang, 2011; Cheng et al., 2012; Luo and Luo, 2012; Lv et al., 2012; Min and Zhang, 2012; Cheng et al., 2013; Mao et al., 2013; Zhao et al., 2013; Yang et al., 2014; Zhao and Jiang, 2014; Yang et al., 2016; Zhai et al., 2016; Hua et al., 2017a; Qian et al., 2018; Yang et al., 2018; Yang et al., 2018; Zhou et al., 2018a; Li et al., 2019b; Qian et al., 2019; Yang et al., 2019; Pan et al., 2021; Shui et al., 2021a; Shui et al., 2021b; Han et al., 2023; Xu et al., 2023; An et al., 2024; Su et al., 2024; Xu et al., 2025; Yang et al., 2025; Zhai et al., 2025a; Zhai et al., 2025b) were rated as high risk due to inherent difficulties in blinding intervention characteristics; remaining studies without relevant reporting were rated as unclear risk.

(3) Detection bias. For random outcome assessment, 33% (n = 25) (Chen et al., 2009b; Lv and Lv, 2009a; Lv and Lv, 2009b; Zhu, 2010; Zou et al., 2010; Zeng et al., 2011; Zeng et al., 2013a; Zeng et al., 2013b; Sun et al., 2014; Zhai et al., 2016; Li et al., 2019a; Si et al., 2020; Li et al., 2025; Yang et al., 2025; Zhai et al., 2025a) explicitly used non-random assessment sequences and were rated as high risk; 67% without specified assessment order were rated as unclear risk. For blinding of outcome assessment, 22% (n = 17) (Meng et al., 2003a; Wu et al., 2006; Chen et al., 2009b; Chen et al., 2010; Qu et al., 2010; Min and Zhang, 2012; Yang et al., 2016; Zhai et al., 2016; Hua et al., 2017a; Qian et al., 2018; Yang et al., 2018; Pan et al., 2021; Han et al., 2023; Su et al., 2024; Yang et al., 2025; Zhai et al., 2025a; Zhai et al., 2025b) failed to implement blinding for subjective outcomes and were rated as high risk; 78% without relevant reporting were rated as unclear risk.

(4) Attrition bias. For incomplete outcome data, 64% (n = 50) (Wang et al., 2004; Chen et al., 2007a; Chen et al., 2007b; Chen et al., 2009a; Chen et al., 2009b; Zou et al., 2010; Cheng et al., 2013; Mao et al., 2013; Zhai et al., 2016; Hua et al., 2017b; Qian et al., 2018; Wang et al., 2018; Li et al., 2019b; Yue et al., 2022; Yang et al., 2025) with complete data were rated as low risk; 18% (n = 14) (Meng et al., 2003b; Luo et al., 2006; Ma et al., 2006; Chen et al., 2007c; Lv and Lv, 2009a; Lv and Lv, 2009b; Zou et al., 2010; Zeng et al., 2011; Luo et al., 2012b; Luo et al., 2012a; Li et al., 2019a; Si et al., 2020; Li et al., 2025) with missing data but reasonable explanations were rated as unclear risk; and 17% (n = 13) (Zeng et al., 2009; Qu et al., 2010; Zhu, 2010; Zeng et al., 2013a; Zeng et al., 2013b; Sun et al., 2014; Lin et al., 2017; Liu and Jin, 2018; Wang et al., 2018; Zhou et al., 2018b; Liu et al., 2021; Liu and Lei, 2022; Liu and Lei, 2024) with unexplained missing data were rated as high risk.

(5) Reporting bias. For selective reporting, 89% (n = 69) (Meng et al., 2003b; Wang et al., 2004; Chen et al., 2007a; Chen et al., 2007b; Chen et al., 2009a; Lv and Lv, 2009a; Zou et al., 2010; Yao and Fang, 2011; Cheng et al., 2012; Lv et al., 2012; Cheng et al., 2013; Mao et al., 2013; Zeng et al., 2013a; Zeng et al., 2013b; Sun et al., 2014; Zhai et al., 2016; Hua et al., 2017b; Hua et al., 2017a; Qian et al., 2018; Wang et al., 2018; Wang et al., 2018; Li et al., 2019a; Li et al., 2019b; Shui et al., 2021a; Yue et al., 2022; Han et al., 2023; Li et al., 2025; Yang et al., 2025) with complete primary outcome reporting were rated as low risk; 9% (n = 7) (Meng et al., 2003a; Chen et al., 2007c; Chen et al., 2009b; Luo et al., 2012b; Luo et al., 2012a; Min and Zhang, 2012; Yang et al., 2025) that could not be judged were rated as unclear risk; and one study (Yi et al., 2020) with missing key outcomes was rated as high risk.

(6) Other bias. No study reported sample size estimation, resulting in unclear risk ratings for all.

The comprehensive risk of bias assessment results across all domains are presented in **Supplementary Table 5**.

### 3.3 Network meta-analysis results

#### 3.3.1 Fatigue-related outcomes

##### 3.3.1.1 Exhaustion time in forced swim test

**Figure 2:**
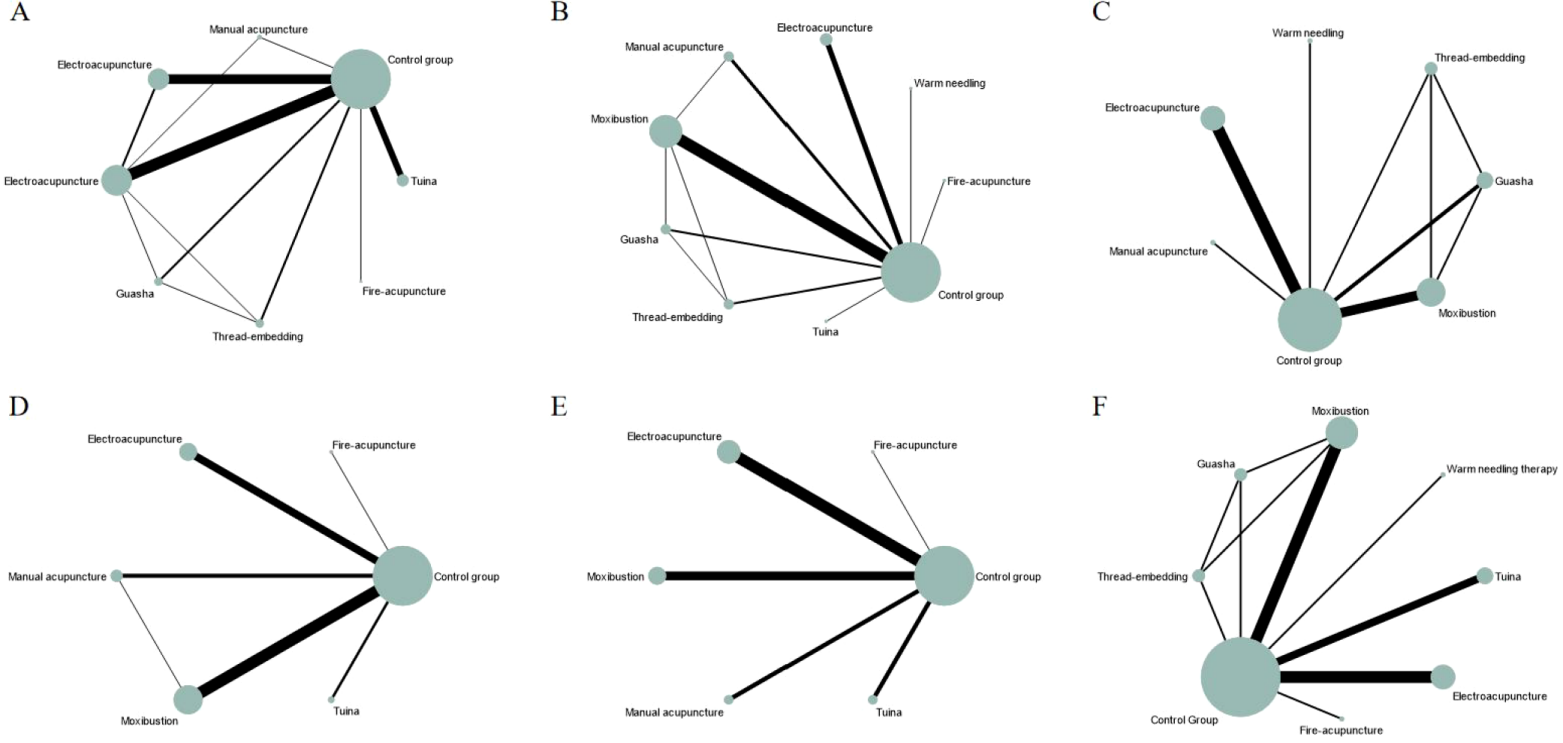
The evidence network for fatigue-related outcomes. **(A)** Exhaustion time in forced swim test; **(B)** Immobility time in tail suspension test; **(C)** Escape latency in morris water maze; **(D)** Vertical score in open field test; **(E)** Horizontal score in open field test; **(F)** Body weight. The thickness of the lines represents the number of studies, and the sizes of the nodes indicate the total sample sizes for each treatment.

Twenty-eight studies (Chen et al., 2007a; Lv and Lv, 2009a; Qu et al., 2010; Zou et al., 2010; Yao and Fang, 2011; Cheng et al., 2012; Luo and Luo, 2012; Cheng et al., 2013c; Zeng et al., 2013a; Sun et al., 2014; Zhai et al., 2016; Hua et al., 2017b; Hua et al., 2017a; Lin et al., 2017; Qian et al., 2018; Wang et al., 2018; Si et al., 2020; Yi et al., 2020; Shui et al., 2021b; Liu and Lei, 2022; Feng et al., 2023; Han et al., 2023; Xu et al., 2023; Liu and Lei, 2024; Lu et al., 2024; Li et al., 2025; Yang et al., 2025) involving 678 experimental animals and seven intervention measures demonstrated that CAM non-pharmacological therapies prolonged exhaustion time in the forced swim test. The network geometry is presented in **Figure 2A**. The inconsistency model test indicated P > 0.05, supporting the use of the consistency model for network meta-analysis. Loop inconsistency tests revealed that the control group formed closed loops with guasha and thread-embedding therapy (IF = 92.372, 95% CI [0, 543.21], P = 0.688), moxibustion and thread-embedding therapy (IF = 83.704, 95% CI [0, 543.57], P = 0.721), electroacupuncture and moxibustion (IF = 44.020, 95% CI [0, 544.48], P = 0.863), moxibustion and guasha (IF = 24.092, 95% CI [0, 523.69], P = 0.925), and moxibustion and manual acupuncture (IF = 14.627, 95% CI [0, 709.68], P = 0.967). Additionally, moxibustion, guasha, and thread-embedding therapy formed a closed loop (IF = 0, 95% CI [0, 3.93], P = 1.000). No statistically significant inconsistency was detected between direct and indirect comparisons (**Table 2**). However, the large absolute IF values suggest a potential trend toward inconsistency, possibly attributable to intervention heterogeneity.

**Table 2:** Summary of loop inconsistency tests across outcome measures.

Outcome measures	Loop	IF	95%CI	P
Exhaustion time in forced swim test	C-GS-TE	92.372	(0, 543.21)	0.688
	C-MB-TE	83.704	(0, 543.57)	0.721
	C-EA-MB	44.020	(0, 544.48)	0.863
	C-MB-GS	24.092	(0, 523.69)	0.925
	C-MB-MA	14.627	(0, 709.68)	0.967
	MB-GS-TE	0	(0, 3.93)	1.000
Immobility time in tail suspension test	C-MB-TE	48.221	(0, 126.32)	0.226
	C-MB-GS	48.218	(0, 126.74)	0.229
	C-MA-MB	16.565	(0, 98.02)	0.690
	C-GS-TE	0	(0, 26.34)	1.000
	MB-GS-TE	0	(0, 25.40)	1.000
Escape latency in morris water maze	C-MB-TE	0.819	(0, 35.57)	0.963
	C-MB-GS	0.702	(0, 26.95)	0.958
	C-GS-TE	0	(0, 16.56)	1.000
	MB-GS-TE	0	(0, 15.72)	1.000
Vertical score in open field test	C-MA-MB	2.471	(0, 15.83)	0.717
Body weight	C-MB-GS	18.338	(0, 51.25)	0.275
	C-MB-TE	18.338	(0, 50.92)	0.270
	MB-GS-TE	0	(0, 16.73)	1.000
	C-GS-TE	0	(0, 16.83)	1.000
IL-1β	C-MA-MB	14.26	(0, 67.59)	0.600
TNF-α	C-MA-MB	48.039	(0, 124.36)	0.217
	C-MB-EA	43.513	(0, 141.48)	0.384
	C-MA-EA	15.547	(0, 108.86)	0.744
	MA-MB-EA	14.784	(0.91, 28.66)	0.037

C, control group; GS, guasha; TE, thread-embedding therapy; MB, moxibustion; EA, electroacupuncture; MA, manual acupuncture.

**Figure 3:**
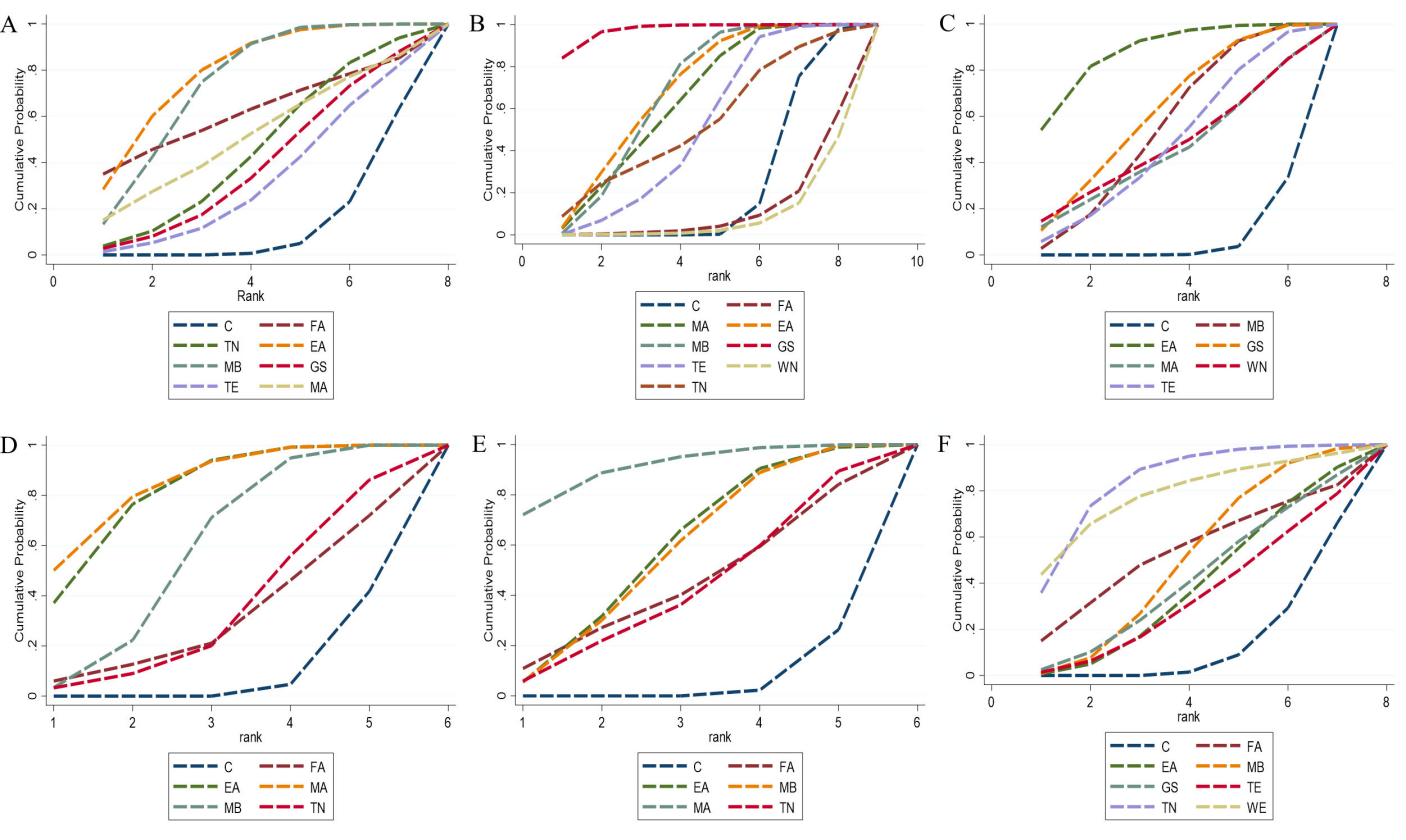
The SUCRA values for fatigue-related outcomes. **(A)** Exhaustion time in forced swim test; **(B)** Immobility time in tail suspension test; **(C)** Escape latency in morris water maze; **(D)** Vertical score in open field test; **(E)** Horizontal score in open field test; **(F)** Body weight. C, control group; MA, manual acupuncture; EA, electroacupuncture; FA, fire-acupuncture; MB, moxibustion; WN, warm needling therapy; TN, tuina; GS, guasha; TE, thread-embedding therapy.

Network meta-analysis demonstrated that all seven intervention measures were superior to the control group in prolonging exhaustion time. Notably, electroacupuncture (MD = 347.00 s, 95% CI [144.70, 549.29]) and moxibustion (MD = 311.28 s, 95% CI [146.36, 476.20]) showed statistically significant differences (P < 0.05). The remaining interventions did not differ significantly from the control group (P > 0.05). Detailed pairwise comparison results are provided in **Supplementary File 6**. According to SUCRA values, electroacupuncture demonstrated the highest probability of being the optimal intervention for prolonging exhaustion time (79.7%), followed by moxibustion (74.4%). SUCRA values for other interventions are presented in **Figure 3A** and **Table 3**.

**Table 3:** The SUCRA values of each treatment modality.

Treatment	SUCRA														
	FST	TST	MWM	OFT-V	OFT-H	BW	SOD	MDA	IL-1β	IL-6	TNF-α	IFN-γ	CRH	ACTH	CORT
Control group	13.2%	23.5%	6.3%	9.3%	5.7%	15.2%	23.6%	0.0%	17.3%	6.0%	44.5%	58.6%	5.5%	10.1%	17.0%
Manual acupuncture	51.9%	64.6%	44.8%	84.5%	91.0%	—	81.6%	25.0%	31.6%	—	53.5%	59.2%	—	—	—
Electroacupuncture	79.7%	69.5%	87.6%	81.3%	58.8%	40.3%	—	—	79.2%	73.4%	57.4%	46.9%	71.6%	47.0%	66.0%
Fire-acupuncture	61.9%	12.0%	—	31.6%	44.4%	53.9%	42.8%	66.9%	—	—	—	—	—	—	—
Moxibustion	74.4%	68.4%	54.9%	58.4%	57.3%	50.8%	—	100.0%	34.7%	41.7%	84.3%	17.2%	34.6%	67.2%	39.3%
Warm needling therapy	—	8.9%	46.8%	—	—	78.6%	—	—	27.4%	28.9%	83.1%	56.8%	—	—	—
Tuina	46.2%	53.7%	—	34.9%	42.7%	84.5%	—	—	100.0%	100.0%	—	—	97.3%	90.4%	93.8%
Guasha	39.6%	97.5%	61.5%	—	—	42.2%	—	—	—	—	12.2%	—	—	—	—
Thread-embedding therapy	33.1%	51.9%	48.1%	—	—	34.6%	—	—	—	—	—	—	41.0%	35.3%	33.9%
Needle-pricking	—	—	—	—	—	—	52.0%	58.1%	59.8%	—	15.1%	61.4%	—	—	—
Best intervention (SUCRA)	Electroacupuncture (79.7%)	Guasha (97.5%)	Electroacupuncture (87.6%)	Manual acupuncture (84.5%)	Manual acupuncture (91.0%)	Tuina (84.5%)	Manual acupuncture (81.6%)	Moxibustion (100%)	Tuina (100%)	Tuina (100%)	Moxibustion (84.3%)	Needle-pricking (61.4%)	Tuina (97.3%)	Tuina (90.4%)	Tuina (93.8%)

FST, exhaustion time in forced swim test; TST, immobility time in tail suspension test; MWM, escape latency in morris water maze; OFT-V, vertical score in open field test; OFT-H, horizontal score in open field test; BW, body weight.

##### 3.3.1.2 Immobility time in tail suspension test

Twenty-three studies (Ma et al., 2006; Chen et al., 2009b; Lv and Lv, 2009a; Zou et al., 2010; Sun et al., 2014; Zhai et al., 2016; Hua et al., 2017b; Hua et al., 2017a; Qian et al., 2018; Feng et al., 2023; Han et al., 2023; Lu et al., 2024; Li et al., 2025; Yang et al., 2025) involving 505 experimental animals and eight intervention strategies examined the effects of complementary and alternative medicine (CAM) non-pharmacological therapies on immobility time in the tail suspension test. The network of interventions is illustrated in **Figure 2B**, with the consistency model employed for network meta-analysis. Compared with the control group, manual acupuncture (MD = -42.31 s, 95% CI [-75.77, -8.85]), electroacupuncture (MD = -46.95 s, 95% CI [-74.52, -19.37]), moxibustion (MD = -44.99 s, 95% CI [-62.08, -27.91]), and guasha (MD = -83.02 s, 95% CI [-115.34, -50.70]) demonstrated statistically significant reductions in immobility time (P < 0.05). No significant differences were observed for the remaining interventions (P > 0.05). Detailed pairwise comparison results are provided in **Supplementary File 6**. Loop inconsistency testing identified five closed loops: control-moxibustion-thread-embedding therapy (IF = 48.221, 95% CI [0, 126.32], P = 0.226), control-moxibustion-guasha (IF = 48.218, 95% CI [0, 126.74], P = 0.229), control-manual acupuncture-moxibustion (IF = 16.565, 95% CI [0, 98.02], P = 0.690), control-guasha-thread-embedding therapy (IF = 0, 95% CI [0, 26.34], P = 1.000), and moxibustion-guasha-thread-embedding therapy (IF = 0, 95% CI [0, 25.40], P = 1.000), indicating no statistically significant inconsistency between direct and indirect evidence (**Table 2**). SUCRA rankings suggested that guasha had the highest probability of being the optimal intervention for reducing immobility time (97.5%), followed by electroacupuncture (69.5%) and moxibustion (68.4%). SUCRA values for other interventions are presented in **Figure 3B** and **Table 3**.

##### 3.3.1.3 Escape latency in Morris water maze

Fourteen studies (Lv and Lv, 2009a; Qu et al., 2010; Luo and Luo, 2012; Cheng et al., 2013; Yang et al., 2018; Zhou et al., 2018b; Li et al., 2019a; Yang et al., 2019; Liu et al., 2021; Shui et al., 2021b; Su et al., 2024; Li et al., 2025; Yang et al., 2025; Zhai et al., 2025b) with 318 experimental animals and six intervention strategies investigated the effects of CAM non-pharmacological therapies on escape latency in the Morris water maze. The network geometry is displayed in **Figure 2C**. Analysis using the consistency model revealed that moxibustion (MD = -17.63 s, 95% CI [-29.05, -6.22]), electroacupuncture (MD = -28.42 s, 95% CI [-39.98, -16.86]), and guasha (MD = -19.47 s, 95% CI [-34.85, -4.09]) significantly shortened escape latency compared with the control group (P < 0.05), whereas other interventions showed no significant differences (P > 0.05). Detailed results are available in **Supplementary File 6**.

Four closed loops were identified: control-moxibustion-thread-embedding therapy (IF = 0.819, 95% CI [0, 35.57], P = 0.963), control-moxibustion-guasha (IF = 0.702, 95% CI [0, 26.95], P = 0.958), control-guasha-thread-embedding therapy (IF = 0, 95% CI [0, 16.56], P = 1.000), and moxibustion-guasha-thread-embedding therapy (IF = 0, 95% CI [0, 15.72], P = 1.000), with no significant inconsistency detected (**Table 2**). According to SUCRA values, electroacupuncture ranked highest for reducing escape latency (87.6%), followed by guasha (61.5%) and moxibustion (54.9%) (**Figure 3C**; **Table 3**).

##### 3.3.1.4 Vertical score in open field test

Twenty studies (Ma et al., 2006; Chen et al., 2007a; Chen et al., 2009b; Zou et al., 2010; Min and Zhang, 2012; Zeng et al., 2013b; Zhai et al., 2016; Hua et al., 2017b; Hua et al., 2017a; Qian et al., 2018; Shui et al., 2021b; Feng et al., 2023; Han et al., 2023; Lu et al., 2024) comprising 456 experimental animals and five intervention strategies evaluated vertical activity scores in the open field test. The network relationships are depicted in **Figure 2D**. Using the consistency model, electroacupuncture (MD = 9.96, 95% CI [5.49, 14.42]), manual acupuncture (MD = 10.51, 95% CI [4.81, 16.21]), and moxibustion (MD = 7.33, 95% CI [3.86, 10.81]) significantly improved vertical scores relative to the control group (P < 0.05). Remaining comparisons were non-significant (P > 0.05). **Supplementary File 6** contains detailed pairwise results. A single closed loop was identified between control-manual acupuncture-moxibustion (IF = 2.471, 95% CI [0, 15.83], P = 0.717), indicating acceptable consistency (**Table 2**). SUCRA analysis indicated manual acupuncture as the most promising intervention for enhancing vertical scores (84.5%), with electroacupuncture (81.3%) and moxibustion (58.4%) following (**Figure 3D** and **Table 3**).

##### 3.3.1.5 Horizontal score in open field test

Twenty-one studies (Chen et al., 2007a; Chen et al., 2009b; Zeng et al., 2009; Qu et al., 2010; Min and Zhang, 2012; Cheng et al., 2013c; Cheng et al., 2013a; Zeng et al., 2013b; Zhai et al., 2016; Hua et al., 2017b; Hua et al., 2017a; Lin et al., 2017; Qian et al., 2018; Yang et al., 2018; Zhou et al., 2018a; Li et al., 2019a; Shui et al., 2021b; Feng et al., 2023; Han et al., 2023; Lu et al., 2024; Xu et al., 2025) involving 480 experimental animals and five intervention strategies assessed horizontal activity scores in the open field test, with the network structure shown in **Figure 2E**. Electroacupuncture (MD = 20.64, 95% CI [9.17, 32.11]), moxibustion (MD = 20.30, 95% CI [7.31, 33.29]), and manual acupuncture (MD = 35.23, 95% CI [14.91, 55.54]) demonstrated significant improvements in horizontal scores compared with controls (P < 0.05). Other interventions did not differ significantly (P > 0.05). See **Supplementary File 6** for complete results. SUCRA rankings identified manual acupuncture as having the greatest potential for improving horizontal scores (91.0%), followed by electroacupuncture (58.8%) and moxibustion (57.3%) (**Figure 3E**; **Table 3**).

##### 3.3.1.6 Body weight

Eighteen studies (Meng et al., 2003a; Wang et al., 2004; Chen et al., 2007a; Lv and Lv, 2009b; Zeng et al., 2009; Yao and Fang, 2011; Cheng et al., 2013c; Zeng et al., 2013a; Yang et al., 2016; Zhai et al., 2016; Hua et al., 2017b; Hua et al., 2017a; Qian et al., 2018; Yang et al., 2018; Yi et al., 2020; Han et al., 2023; Xu et al., 2023; Li et al., 2025) with 403 experimental animals and seven intervention strategies examined body weight changes, with the intervention network presented in **Figure 2F**. Only tuina (MD = 43.61 g, 95% CI [13.42, 73.80]) showed a statistically significant increase in body weight relative to the control group (P < 0.05); other comparisons were non-significant (P > 0.05). Detailed results are provided in **Supplementary File 6**. Loop inconsistency testing revealed four closed loops: control-moxibustion-guasha (IF = 18.338, 95% CI [0, 51.25], P = 0.275), control-moxibustion-thread-embedding therapy (IF = 18.338, 95% CI [0, 50.92], P = 0.270), moxibustion-guasha-thread-embedding therapy (IF = 0, 95% CI [0, 16.73], P = 1.000), and control-guasha-thread-embedding therapy (IF = 0, 95% CI [0, 16.83], P = 1.000), with no significant inconsistency between direct and indirect evidence (**Table 2**). SUCRA values suggested tuina as the most effective intervention for increasing body weight (84.5%), followed by warm needling therapy (78.6%) and fire acupuncture (53.9%) (**Figure 3F**; **Table 3**).

#### 3.3.2 Oxidative stress-related outcomes

##### 3.3.2.1 SOD activity

**Figure 4:**
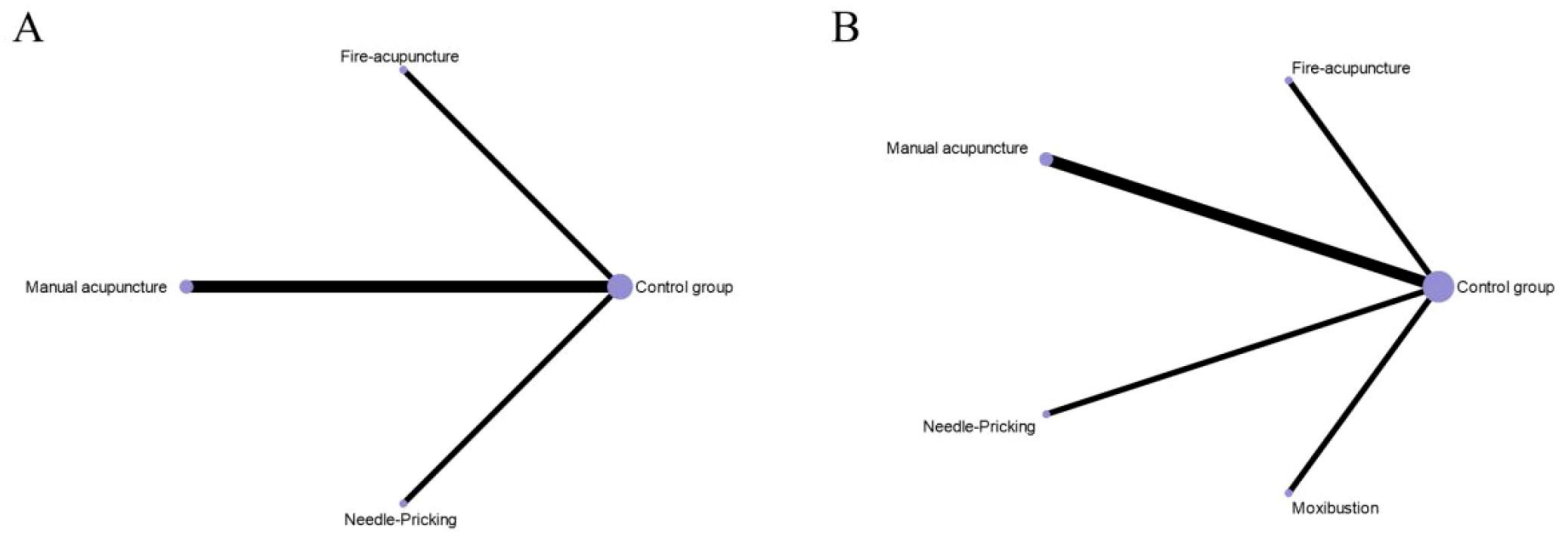
The evidence network for oxidative stress-related outcomes. **(A)** SOD level; **(B)** MDA level. The thickness of the lines represents the number of studies, and the sizes of the nodes indicate the total sample sizes for each treatment.

**Figure 5:**
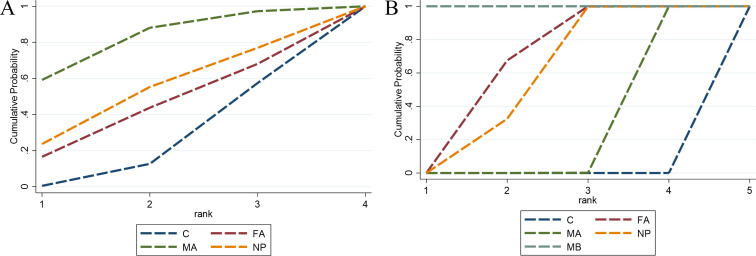
The SUCRA values for oxidative stress-related outcomes. **(A)** SOD level; **(B)** MDA level. C, control group; MA, manual acupuncture; FA, fire-acupuncture; MB, moxibustion; NP, needle-pricking.

Four studies (Luo et al., 2006; Chen et al., 2009b; Chen et al., 2010; Qian et al., 2018) involving 81 experimental animals and three intervention measures demonstrated that complementary and alternative medicine (CAM) non-pharmacological therapies could enhance superoxide dismutase (SOD) activity to a certain extent, with the network of interventions illustrated in **Figure 4A**. The inconsistency model test yielded P > 0.05, supporting the suitability of the consistency model for network meta-analysis. All three interventions showed advantages over the control group in improving SOD activity, though none reached statistical significance (P > 0.05), with detailed pairwise comparison results provided in **Supplementary File 6**. According to SUCRA rankings, manual acupuncture exhibited greater efficacy in enhancing SOD activity (81.6%), followed by needle-pricking therapy (52.0%), with SUCRA values for other interventions presented in **Figure 5A** and **Table 3**.

##### 3.3.2.2 MDA concentration

Five studies (Luo et al., 2006; Chen et al., 2009b; Chen et al., 2010; Qian et al., 2018; Zhai et al., 2025b) comprising 101 experimental animals and four intervention measures demonstrated reductions in malondialdehyde (MDA) concentration following CAM non-pharmacological therapies, with network relationships depicted in **Figure 4B**. The inconsistency model test indicated P > 0.05, confirming the appropriateness of the consistency model. All four interventions showed statistically significant advantages over the control group in reducing MDA concentration (P < 0.05): fire acupuncture (MD = -13.15 nmol/ml, 95% CI [-18.06, -8.24]), manual acupuncture (MD = -3.85 nmol/ml, 95% CI [-5.12, -2.58]), needle-pricking therapy (MD = -11.43 nmol/ml, 95% CI [-16.54, -6.32]), and moxibustion (MD = -479.16 nmol/ml, 95% CI [-567.05, -391.27]), and detailed results are available in **Supplementary File 6**. SUCRA analysis identified moxibustion as having the greatest efficacy in reducing MDA concentration (100.0%), with fire acupuncture also showing substantial effects (66.9%), as illustrated in **Figure 5B** and **Table 3**.

#### 3.3.3 Inflammation-related outcomes

##### 3.3.3.1 IL-1β level

**Figure 6:**
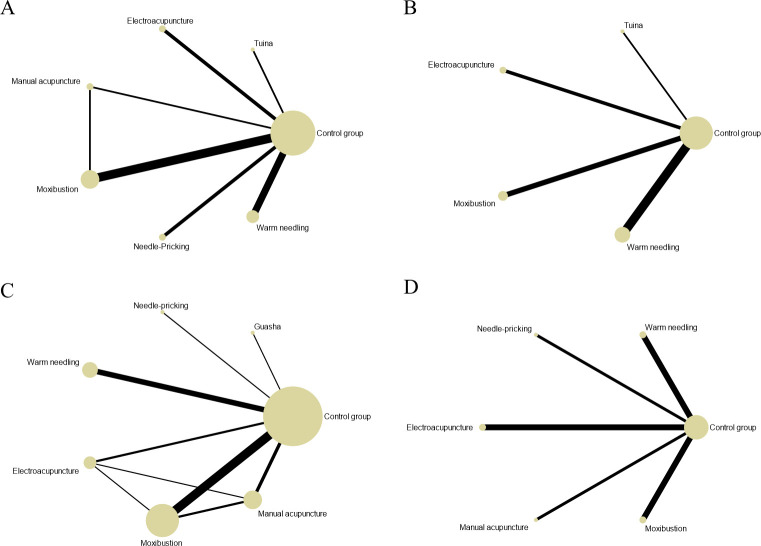
The evidence network for inflammation-related outcomes. **(A)** IL-1βlevel; **(B)** IL-6 level; **(C)** TNF-α; **(D)** IFN-γ. The thickness of the lines represents the number of studies, and the sizes of the nodes indicate the total sample sizes for each treatment.

**Figure 7:**
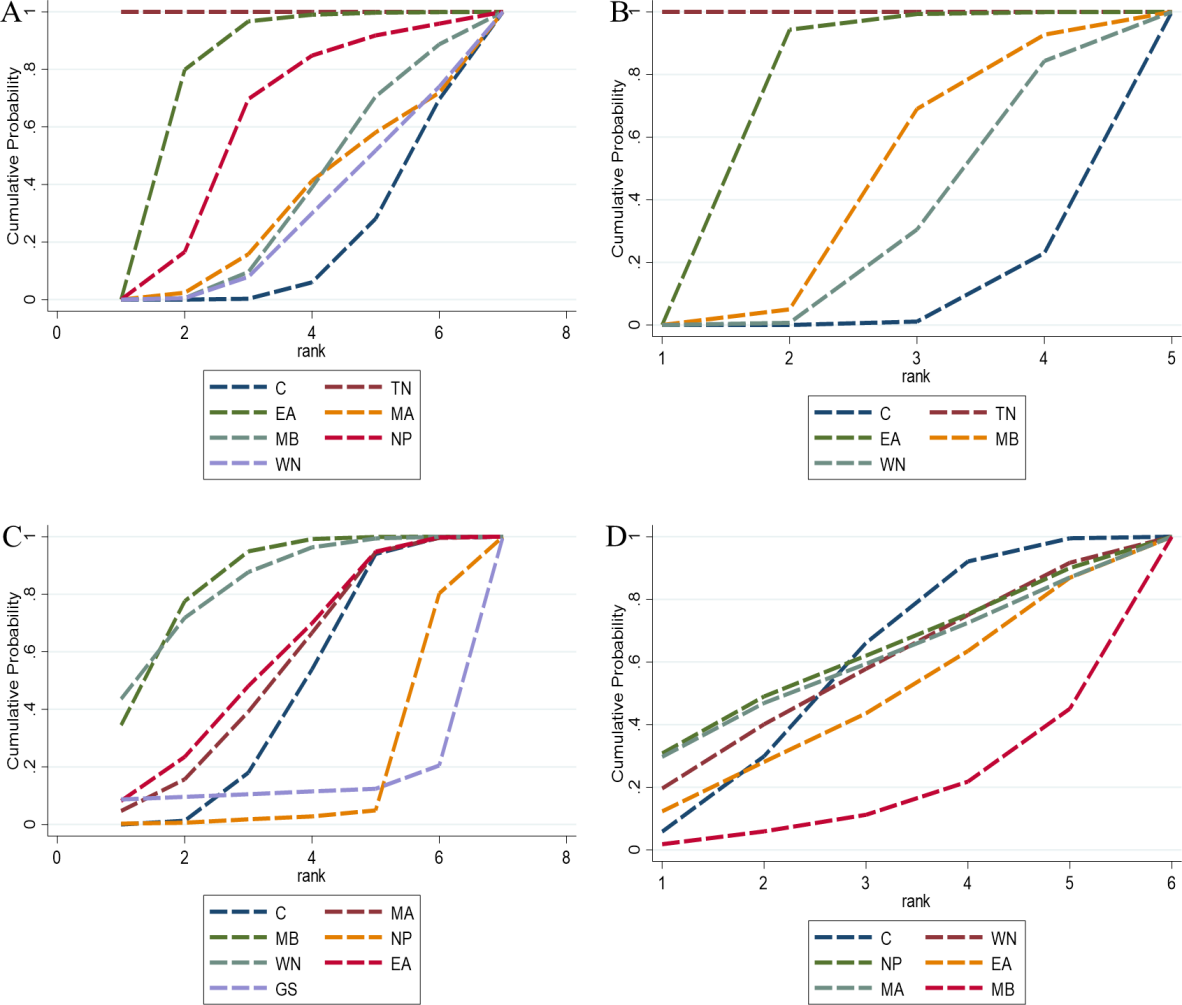
The SUCRA values for inflammation-related outcomes. **(A)** IL-1βlevel; **(B)** IL-6 level; **(C)** TNF-α; **(D)** IFN-γ. C, control group; MA, manual acupuncture; EA, electroacupuncture; MB, moxibustion; WN, warm needling therapy; NP, needle-pricking.

Eleven studies (Chen et al., 2007a; Chen et al., 2007c; Chen et al., 2010; Zeng et al., 2011; Luo et al., 2012b; Zeng et al., 2013a; Yang et al., 2014; Yang et al., 2016; Wang et al., 2018; Su et al., 2024; Zhai et al., 2025a) involving 259 experimental animals and six intervention measures demonstrated that complementary and alternative medicine (CAM) non-pharmacological therapies reduced interleukin-1β (IL-1β) levels compared with the control group, with the network of interventions illustrated in **Figure 6A**. The inconsistency model test yielded P > 0.05, supporting the suitability of the consistency model for network meta-analysis. All six interventions showed advantages over the control group in reducing IL-1β levels, with tuina (MD = -552.03 pg/ml, 95% CI [-700.81, -403.25]) and electroacupuncture (MD = -156.59 pg/ml, 95% CI [-259.85, -53.34]) demonstrating statistically significant differences (P < 0.05), whereas remaining comparisons were non-significant (P > 0.05), and detailed pairwise results are provided in **Supplementary File 6**. Loop inconsistency testing identified a closed loop among control-manual acupuncture-moxibustion (IF = 14.26, 95% CI [0, 67.59], P = 0.600), indicating no statistically significant inconsistency between direct and indirect evidence (**Table 2**). According to SUCRA rankings, tuina exhibited the greatest efficacy in reducing IL-1β levels (100.0%), followed by electroacupuncture (79.2%), with SUCRA values for other interventions presented in **Figure 7A** and **Table 3**.

##### 3.3.3.2 IL-6 level

Eight studies (Zeng et al., 2011; Zeng et al., 2013a; Yang et al., 2014; Yang et al., 2016; Wang et al., 2018; Yi et al., 2020; Su et al., 2024; Zhai et al., 2025a) comprising 181 experimental animals and four intervention measures examined the effects of CAM non-pharmacological therapies on interleukin-6 (IL-6) levels, with network relationships depicted in **Figure 6B**. The inconsistency model test indicated P > 0.05, confirming the appropriateness of the consistency model. All four interventions demonstrated advantages over the control group in reducing IL-6 levels, notably tuina (MD = -554.77 pg/ml, 95% CI [-693.88, -415.66]) and electroacupuncture (MD = -163.27 pg/ml, 95% CI [-260.27, -66.27]) showed statistically significant differences (P < 0.05), while other comparisons did not reach significance (P > 0.05), with detailed results available in **Supplementary File 6**. SUCRA analysis identified tuina as having the highest probability of being the optimal intervention for reducing IL-6 levels (100.0%), with electroacupuncture also showing substantial effects (73.4%), as illustrated in **Figure 7B** and **Table 3**.

##### 3.3.3.3 TNF-α level

Fourteen studies (Chen et al., 2007c; Chen et al., 2010; Zou et al., 2010; Luo et al., 2012b; Lv et al., 2012; Cheng et al., 2013b; Yang et al., 2014; Yang et al., 2016; Li et al., 2019b; Yang et al., 2019; Yi et al., 2020; Liu and Lei, 2024; Su et al., 2024; Zhai et al., 2025a) with 281 experimental animals and six intervention measures were included, with the intervention network presented in **Figure 6C**. The inconsistency model test produced P > 0.05, establishing the consistency model as appropriate for analysis. Network meta-analysis revealed that manual acupuncture, moxibustion, warm needling therapy, and electroacupuncture reduced tumor necrosis factor-α (TNF-α) levels, whereas needle-pricking therapy and guasha increased TNF-α levels, though none of these differences achieved statistical significance (P > 0.05), and **Supplementary File 6** contains detailed pairwise comparisons. Loop inconsistency testing identified four closed loops: control-manual acupuncture-moxibustion (IF = 48.039, 95% CI [0, 124.36], P = 0.217), control-moxibustion-electroacupuncture (IF = 43.513, 95% CI [0, 141.48], P = 0.384), control-manual acupuncture-electroacupuncture (IF = 15.547, 95% CI [0, 108.86], P = 0.744), and manual acupuncture-moxibustion-electroacupuncture (IF = 14.784, 95% CI [0.91, 28.66], P = 0.037), with no statistically significant inconsistency between direct and indirect evidence (**Table 2**). According to SUCRA values, moxibustion demonstrated the greatest efficacy in reducing TNF-α levels (84.3%), followed closely by warm needling therapy (83.1%), as shown in **Figure 7C** and **Table 3**.

##### 3.3.3.4 IFN-γ level

Eight studies (Zhang et al., 2015; Meng et al., 2003a; Tang et al., 2007; Chen et al., 2010; Zhu, 2010; Yang et al., 2014; Yang et al., 2019; Liu and Lei, 2024) involving 172 experimental animals and five intervention measures were incorporated, with network geometry displayed in **Figure 6D**. The inconsistency model test generated P > 0.05, supporting the use of the consistency model. Network meta-analysis indicated that warm needling therapy, electroacupuncture, and moxibustion reduced interferon-γ (IFN-γ) levels, while needle-pricking therapy and manual acupuncture increased IFN-γ levels, though none of these effects were statistically significant (P > 0.05), and complete results are provided in **Supplementary File 6**. SUCRA rankings suggested needle-pricking therapy had the highest probability of increasing IFN-γ levels (61.4%), with SUCRA values for other interventions presented in **Figure 7D** and **Table 3**.

#### 3.3.4 Endocrine-related outcomes

##### 3.3.4.1 CRH level

**Figure 8:**
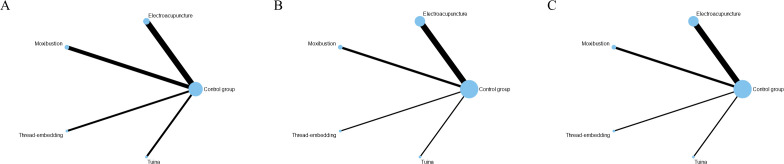
The evidence network for endocrine-related outcomes. **(A)** CRH level; **(B)** ACTH level; **(C)** CORT level. The thickness of the lines represents the number of studies, and the sizes of the nodes indicate the total sample sizes for each treatment.

**Figure 9:**
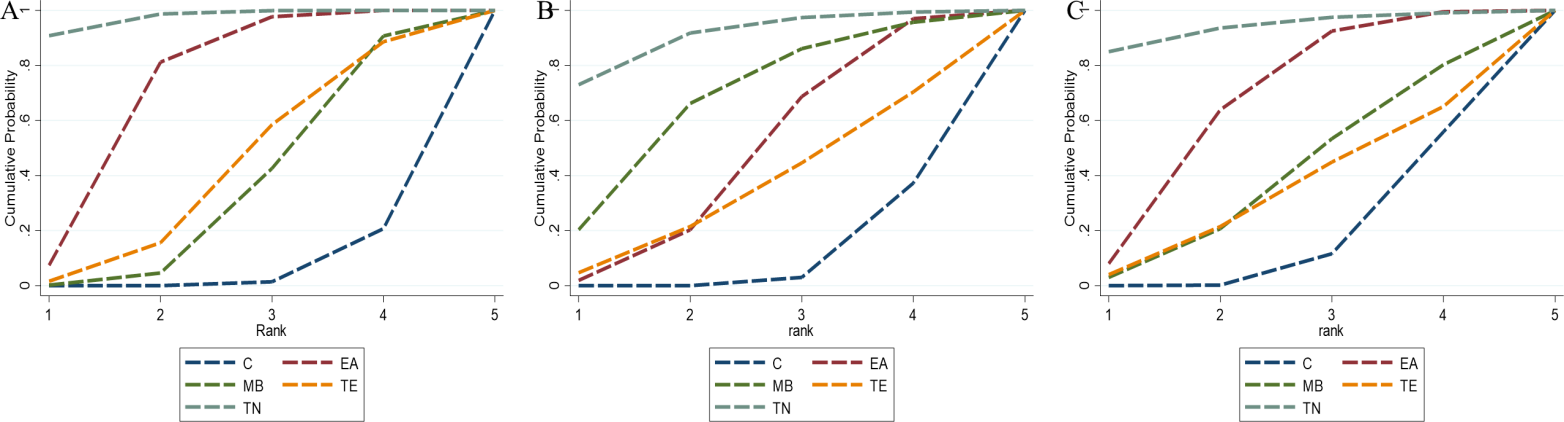
The SUCRA values for endocrine-related outcomes. **(A)** CRH level; **(B)** ACTH level; **(C)** CORT level. C, control group; EA, electroacupuncture; MB, moxibustion; TN, tuina; TE, thread-embeddingtherapy.

Seven studies (Lv and Lv, 2009a; Zeng et al., 2013b; Zhai et al., 2016; Yang et al., 2018; Qian et al., 2019; Si et al., 2020; Shui et al., 2021b) involving 154 experimental animals and four intervention measures demonstrated that complementary and alternative medicine (CAM) non-pharmacological therapies reduced corticotropin-releasing hormone (CRH) levels, with the network of interventions illustrated in **Figure 8A**. The inconsistency model test yielded P > 0.05, supporting the suitability of the consistency model for network meta-analysis. All four interventions showed advantages over the control group in reducing CRH levels, with electroacupuncture (MD = -9.91 pg/ml, 95% CI [-14.77, -5.05]) and tuina (MD = -16.96 pg/ml, 95% CI [-25.37, -8.55]) demonstrating statistically significant differences (P < 0.05), whereas remaining comparisons were non-significant (P > 0.05), and detailed pairwise results are provided in **Supplementary File 6**. According to SUCRA rankings, tuina exhibited the greatest efficacy in reducing CRH levels (97.3%), followed by electroacupuncture (71.6%), with SUCRA values for other interventions presented in **Figure 9A** and **Table 3**.

##### 3.3.4.2 ACTH level

Nine studies (Wang et al., 2004; Lv and Lv, 2009a; Qu et al., 2010; Cheng et al., 2013; Zeng et al., 2013b; Zhai et al., 2016; Yang et al., 2018; Si et al., 2020; Shui et al., 2021b) comprising 207 experimental animals and four intervention measures examined the effects of CAM non-pharmacological therapies on adrenocorticotropic hormone (ACTH) levels, with network relationships depicted in **Figure 8B**. The inconsistency model test indicated P > 0.05, confirming the appropriateness of the consistency model. All four interventions demonstrated advantages over the control group in reducing ACTH levels, notably tuina (MD = -20.02 pg/ml, 95% CI [-36.11, -3.93]) showed statistically significant differences (P < 0.05), while other comparisons did not reach significance (P > 0.05), with detailed results available in **Supplementary File 6**. SUCRA analysis identified tuina as having the highest probability of being the optimal intervention for reducing ACTH levels (90.4%), with moxibustion also showing substantial effects (67.2%), as illustrated in **Figure 9B** and **Table 3**.

##### 3.3.4.2 CORT level

Nine studies (Wang et al., 2004; Lv and Lv, 2009a; Qu et al., 2010; Cheng et al., 2013; Zeng et al., 2013b; Zhai et al., 2016; Yang et al., 2018; Si et al., 2020; Shui et al., 2021b) with 207 experimental animals and four intervention measures provided evidence that CAM non-pharmacological therapies downregulated cortisol (CORT) levels in fatigue-like condition animal models, with the intervention network presented in **Figure 8C**. The inconsistency model test produced P > 0.05, establishing the consistency model as appropriate for analysis. Network meta-analysis revealed that all four interventions showed advantages over the control group in reducing CORT levels, with electroacupuncture (MD = -35.69 ng/ml, 95% CI [-65.43, -5.95]) and tuina (MD = -89.95 ng/ml, 95% CI [-170.13, -9.77]) demonstrating statistically significant differences (P < 0.05), whereas remaining comparisons were non-significant (P > 0.05), and **Supplementary File 6** contains detailed pairwise comparisons. According to SUCRA values, tuina demonstrated the greatest efficacy in reducing CORT levels (93.8%), followed by electroacupuncture (66.0%), as shown in **Figure 9C** and **Table 3**.

#### 3.3.5 Assessment of small-study effects

Comparison-adjusted funnel plots for body weight, immobility time in tail suspension test, escape latency in Morris water maze, vertical score in open field test, IL-1β, IL-6, and CORT demonstrated approximately symmetrical distribution of study points around the null line, suggesting low likelihood of publication bias in these outcomes. Conversely, funnel plots for exhaustion time in forced swim test, horizontal score in open field test, SOD activity, MDA concentration, TNF-α, IFN-γ, ACTH, and CRH displayed asymmetry, indicating potential publication bias or small-study effects. These findings are presented in **Supplementary File 7**.

#### 3.3.6 *Post-hoc* subgroup analysis by modeling method

Given the heterogeneity across animal models and the distinction between human CFS and fatigue-like condition models, we conducted an exploratory *post-hoc* subgroup analysis to examine whether the relative efficacy of CAM non-pharmacological therapies differs according to the method used to induce fatigue-like conditions. This analysis was not pre-specified in the PROSPERO protocol (CRD420251177070) and should be interpreted as hypothesis-generating. Based on the classification framework proposed by Feng et al (Feng et al., 2025), the included animal models were categorized into three major types according to their induction mechanisms: physical stress models, immune-neuroendocrine stress models, and psychological stress models. Specifically, physical stress models included forced swimming and treadmill training; immune-neuroendocrine stress models included lipopolysaccharide (LPS) injection, Brucella abortus injection, poly I:C injection, and adrenalectomy; psychological stress models included chronic restraint, sleep deprivation, tail clamping, tail suspension, crowded environment, and food restriction. Subgroup analyses revealed that for the outcomes of exhaustion time in forced swim test, immobility time in tail suspension test, escape latency in Morris water maze, vertical score in open field test, horizontal score in open field test, IFN-γ, CRH, ACTH and CORT, the subgroup results were consistent with the overall analysis, indicating robust findings. Conversely, for body weight, IL-1β, IL-6 and TNF-α, the subgroup results diverged from the overall analysis, suggesting that modeling methods may influence these particular outcomes. These findings are presented in **Supplementary Table 8**.

## 4 Discussion

### 4.1 Summary of main results

This study represents the first network meta-analysis comparing the multidimensional efficacy of nine CAM non-pharmacological therapies—manual acupuncture, electroacupuncture, fire acupuncture, moxibustion, warm needling therapy, tuina, guasha, thread-embedding therapy, and needle-pricking therapy—in animal models of fatigue-like conditions. The results demonstrated that manual acupuncture and electroacupuncture exhibited significant efficacy in ameliorating fatigue levels; moxibustion was most effective in reducing systemic oxidative stress markers; and tuina showed the greatest efficacy in alleviating inflammatory states and regulating endocrine parameters. It is crucial to acknowledge that the animal models included in this study—primarily induced by forced swimming, sleep deprivation, or dietary restriction—reproduce fatigue-like behaviors and biochemical changes but do not fully capture the complex, multi-system pathophysiology of human CFS. Specifically, these models cannot replicate the post-exertional malaise (PEM), which is a pathognomonic feature of CFS, nor the chronic, relapsing-remitting course of the human disease. Therefore, our findings should be interpreted as demonstrating effects on fatigue-related phenotypes rather than direct therapeutic efficacy against CFS itself.

### 4.2 Interpretation of results and mechanistic exploration

The outcome measures of this study encompass four domains: fatigue level, oxidative stress, inflammatory markers, and endocrine parameters. These findings provide insights into potential mechanisms underlying fatigue-related pathologies.

Six outcome measures reflected fatigue levels. Exhaustion time in forced swim test indicates overall physical fitness and skeletal muscle endurance, with reduced time representing the most direct behavioral evidence of physical fatigue; electroacupuncture ranked highest for prolonging exhaustion time (SUCRA = 79.7%). Immobility time in tail suspension test reflects the degree of behavioral despair in mice, with longer duration indicating higher despair levels; guasha ranked highest for reducing immobility time (SUCRA = 97.5%). Escape latency in Morris water maze reflects spatial learning and memory capabilities, with prolonged latency indicating decreased cognitive processing speed due to fatigue; electroacupuncture ranked highest for reducing escape latency (SUCRA = 87.6%). Horizontal and vertical scores in open field test quantify exploratory activity in experimental animals, with their combination enabling determination of fatigue type; manual acupuncture ranked highest for improving both vertical (SUCRA = 84.5%) and horizontal scores (SUCRA = 91.0%). Body weight serves as an objective indicator of systemic debility; tuina ranked highest for increasing body weight (SUCRA = 84.5%). Collectively, electroacupuncture and manual acupuncture demonstrated advantages over other interventions in ameliorating fatigue levels.

Two outcome measures reflected oxidative stress: SOD activity and MDA concentration. Regarding SOD activity, none of the interventions showed statistically significant differences compared with the control group. MDA concentration directly reflects the degree of oxidative stress-induced damage; elevated MDA indicates attack and destruction of membrane unsaturated fatty acids by reactive oxygen species, reflecting antioxidant defense system imbalance. In chronic fatigue models, increased MDA is closely associated with mitochondrial dysfunction, energy metabolism disturbances, and persistent physical fatigue, serving as a classic marker for assessing oxidative damage severity. For reducing MDA concentration, moxibustion ranked highest (SUCRA = 84.5%). This finding differs from the conclusion that electroacupuncture and manual acupuncture demonstrate superior efficacy in ameliorating fatigue levels. The underlying mechanisms may be explained as follows: moxibustion employs thermal stimulation generated by burning mugwort wool as its core intervention, comprising synergistic thermal-photon-pharmacological effects. Local temperatures can reach 45–50 °C, and this controlled thermal stress activates heat shock protein expression (HSP70, HSP90), enhancing cellular tolerance to oxidative damage (Chang et al., 2006). Simultaneously, thermal stimulation promotes local vasodilation and microcirculatory improvement, accelerating metabolic waste clearance and reducing free radical accumulation (Zhang et al., 2022). In contrast, manual acupuncture primarily relies on mechanical stimulation, which may have limited breadth and depth in counteracting oxidative stress. Notably, animal experimental studies investigating electroacupuncture’s effects on oxidative stress damage in fatigue-like condition models are currently lacking and warrant further validation.

Four outcome measures reflected inflammatory status: IL-1β, IL-6, TNF-α, and IFN-γ levels. For TNF-α and IFN-γ levels, none of the interventions demonstrated statistically significant differences compared with the control group. IL-1β serves as an upstream trigger initiating inflammatory responses, while IL-6 maintains and amplifies this signal, collectively leading to peripheral tissue energy metabolism disturbances and central motivation system suppression. For reducing IL-1β and IL-6 levels, tuina ranked highest (SUCRA = 100.0% and 100.0%, respectively). This finding diverges from the conclusion that electroacupuncture and manual acupuncture exhibit superior efficacy in ameliorating fatigue levels. The underlying mechanisms may be explained as follows: tuina applies sustained mechanical pressure to the body surface through manual techniques, with a substantially larger area of effect than single-point acupuncture, simultaneously covering muscle, fascia, subcutaneous tissue, and deep structures. This broad-range mechanical stimulation comprehensively activates mechanosensitive ion channels (e.g., Piezo1, Piezo2) in tissues, converting physical stimuli into intracellular biochemical cascades via the cAMP-Epac1-Rap1 signaling axis, directly inhibiting the TLR4/NF-κB inflammatory pathway and downregulating pro-inflammatory cytokine expression including IL-1β and IL-6 (Liu et al., 2021). In contrast, although manual acupuncture and electroacupuncture can activate local mechanoreceptors, their stimulation range is limited. Furthermore, if electrical parameters (frequency, intensity) are not optimized, electroacupuncture may induce minor tissue damage due to excessive stimulation, triggering compensatory inflammatory responses. Notably, IL-1β was included in 16 studies and IL-6 in 11 studies. According to the Cochrane Handbook, heterogeneity tests and consistency assessments have substantially reduced power with limited numbers of studies. Therefore, SUCRA rankings for these outcomes should be considered exploratory hypotheses and interpreted with caution.

Three outcome measures reflected endocrine parameters: CRH, ACTH, and CORT levels. Effective complementary and alternative medicine therapies should restore circadian rhythms of the HPA axis and enhance stress response flexibility. According to SUCRA rankings, tuina ranked highest in regulating HPA axis circadian rhythms. This finding differs from the conclusion that electroacupuncture and manual acupuncture demonstrate superior efficacy in improving overall fatigue levels. The underlying mechanisms may be explained as follows: tuina’s broad-range somatosensory input activates the parasympathetic nervous system and reduces sympathetic tone (Field, 2016); direct mechanical stimulation of cervical, thoracic, and dorsal regions where the vagus nerve is distributed can rapidly inhibit CRH neuronal activity via the vagus nerve-hypothalamus pathway (Kurhaluk et al., 2025); enhanced local blood circulation and lymphatic return accelerate clearance of peripheral glucocorticoid metabolites, alleviating sustained HPA axis activation; and oxytocin release induced by somatic contact directly inhibits stress hormone secretion (Uvnäs-Moberg et al., 2014). In comparison, electroacupuncture’s stimulation is confined to local acupoint areas, its invasive electrical properties may induce minor stress responses, and the lack of interpersonal thermal contact results in weaker oxytocin activation effects, rendering it slightly less effective in overall HPA axis modulation. Notably, original animal experimental studies investigating manual acupuncture’s effects on endocrine parameters in these models are currently lacking and warrant further validation.

Although the top-ranked interventions differ between macroscopic and microscopic levels, electroacupuncture and manual acupuncture demonstrated statistically significant differences compared with the control group for most indicators and achieved favorable rankings, suggesting that these modalities may improve macroscopic fatigue severity in fatigue-like condition animal models through combined micro-mechanisms including ameliorating oxidative stress, reducing inflammatory damage, and modulating HPA axis function. This hypothesis requires further investigation. Additionally, comparative studies examining efficacy differences between manual acupuncture and electroacupuncture are warranted.

### 4.3 Comparison with other studies

Compared with existing evidence, this study represents the first systematic evaluation of CAM non-pharmacological therapies as an integrated category in animal models of CFS, thereby addressing a critical gap in this field. Previous literature has predominantly focused on exercise (Liao et al., 2025; Wei et al., 2025) or cognitive behavioral interventions (Kolala et al., 2025), or assessed single modalities such as acupuncture or massage in isolation (Feng et al., 2025; Li et al., 2024). In contrast, this network meta-analysis simultaneously encompasses nearly all mainstream CAM non-pharmacological approaches—including manual acupuncture, electroacupuncture, fire acupuncture, moxibustion, warm needling therapy, tuina, guasha, thread-embedding therapy, and needle-pricking therapy—substantially broadening the intervention spectrum. Furthermore, existing evidence has primarily relied on subjective endpoints based on clinical scales; for instance, the study by Feng Chuwen et al (Feng et al., 2025). focused on the Fatigue Severity Scale, Somatic and Psychological Health Report, and Self-Rating Depression Scale, lacking reproducible objective biomarkers and thereby limiting the depth of mechanistic exploration. By utilizing animal experiments, this study integrates multidimensional indicators spanning behavioral performance, oxidative stress, inflammatory markers, and endocrine parameters, providing a quantifiable biological evidence chain for subsequent translational research. Additionally, our ranking results align directionally with conclusions from previous single-therapy meta-analyses, further reinforcing the feasibility and reproducibility of CAM non-pharmacological interventions in preclinical CFS models.

### 4.4 Limitations and strengths

#### 4.4.1 Limitations

This study has several limitations. Although no language restrictions were applied during literature retrieval, the searched databases were predominantly English and Chinese; consequently, only studies published in these two languages were ultimately included, potentially omitting evidence in other languages. Grey literature such as conference abstracts and dissertations were excluded due to concerns regarding data quality and reproducibility, which may have led to overestimation of effect sizes. Publication bias resulting from restricted dissemination of negative results further compromised the accuracy of effect estimates. Regarding animal models, existing CFS models (forced swimming, combined stress, sleep deprivation, etc.) cannot fully replicate the complex pathophysiology of multi-system interactions in humans, necessitating cautious extrapolation. Furthermore, none of the included models replicate the post-exertional malaise characteristic of human CFS, representing a fundamental limitation in the translational validity of these findings. Original experiments varied substantially in animal strains, modeling methods, intervention parameters (intensity, frequency, duration), and outcome measures; the resulting heterogeneity in baseline fatigue levels may have inflated pooled error. Additionally, the absence of head-to-head direct comparisons increased uncertainty in network meta-analysis estimates. Constrained by the depth of original literature, mechanistic interpretations in this study remain largely inferential and require targeted basic experiments for validation.

#### 4.4.2 Strengths

Methodologically, this study strictly adhered to PRISMA-NMA guidelines and was prospectively registered on an open science platform, ensuring traceable protocols and reproducible results. Risk of bias in animal experiments was systematically assessed using the SYRCLE tool. The search strategy and eligibility criteria were finalized after multiple rounds of deliberation. Comprehensive searches were conducted across eight authoritative databases (CNKI, Wanfang, VIP, SinoMed, PubMed, Embase, Web of Science, and Cochrane Library). The entire process—from literature screening to data extraction and quality assessment—was completed by two researchers following double-blind, independent, cross-checking principles, minimizing selection and reporting bias. In terms of content, this study represents the first network meta-analysis of multiple CAM non-pharmacological therapies in fatigue-like condition animal models, quantitatively comparing efficacy differences across fatigue level, oxidative stress, inflammation, and endocrine parameters, thereby filling an evidence gap in this field. Its preclinical value lies not only in identifying the most promising interventions but also in providing solid experimental foundations and testable scientific hypotheses for target selection, dose determination, and mechanistic validation in subsequent human clinical trials.

### 4.5 Implications for future research

Future research should prioritize standardization of fatigue-like condition animal models by establishing a multimodal core outcome set encompassing behavioral performance, oxidative stress, inflammation, and endocrine parameters, while harmonizing animal strains, sex, age, and comorbidity backgrounds to enhance cross-experimental comparability and external validity. For manual acupuncture, electroacupuncture, moxibustion, and tuina—which ranked highest in this study—rigorous head-to-head randomized controlled experiments should be conducted under standardized parameters, integrating proteomics and metabolomics to deeply parse molecular mechanisms underlying differential effects, and further exploring dose-response curves as well as sex-age interactions in female, aged, and anxiety-depression comorbidity contexts. Based on thus strengthened preclinical evidence, subsequent human clinical trials may prioritize evaluating “manual acupuncture/electroacupuncture ± moxibustion” and “tuina” protocols using adaptive platform designs, simultaneously collecting peripheral oxidative, inflammatory, and HPA axis biomarkers alongside subjective fatigue scales to achieve seamless bridging of animal-human biomarkers. Under current evidence gradients, clinicians may consider the aforementioned therapies as priority options for CFS adjunctive interventions, provided patient preferences and resource accessibility are adequately weighed, with continuous real-world tracking of safety and long-term efficacy to progressively elevate evidence levels.

## 5 Conclusion

This study represents the first application of network meta-analysis to systematically compare the intervention effects of nine CAM non-pharmacological therapies in animal models of fatigue-like conditions. The findings indicate that manual acupuncture and electroacupuncture demonstrate significant efficacy in ameliorating fatigue levels; moxibustion excels in reducing systemic oxidative stress markers; and tuina proves most effective in alleviating inflammatory states and regulating endocrine parameters. Collectively, different CAM non-pharmacological therapies exhibit distinct advantages across various dimensions of fatigue-like conditions. Preclinical evidence supports electroacupuncture, manual acupuncture, moxibustion, and tuina as multi-target interventions with substantial translational potential. Future research should prioritize methodologically rigorous, large-scale animal experiments targeting key pathological mechanisms, alongside well-designed clinical trials to validate their safety and efficacy. Additionally, individualized and combined acupoint intervention strategies warrant further exploration to provide evidence-based complementary therapeutic options for CFS patients.

## Data Availability

The original contributions presented in the study are included in the article/**Supplementary Material**. Further inquiries can be directed to the corresponding authors.
