# Supplementary material for: Effectiveness of non-pharmacological therapies in complementary and alternative medicine on improving fatigue levels, oxidative stress, inflammation, and endocrine levels in animal models of chronic fatigue-like conditions: a systematic review and network meta-analysis
Source: Front Physiol. 2026 May 7;17:1807587. doi: 10.3389/fphys.2026.1807587 (PMC13189903; doi:10.3389/fphys.2026.1807587)
Supplement: Supplementary file 1 [file DataSheet1.docx]

**Contents**

[Supplementary File 1 Database search strategy 1](#_Toc29888)

[Supplementary File 2 Conversion formula for standard deviation (SD). 4](#_Toc23671)

[Supplementary Table S3 Definition of interventions and controls. 5](#_Toc9917)

[Supplementary Table S4 Basic characteristics of the included literatures 7](#_Toc30938)

[Supplementary Table S5 Results of risk of bias assessment using SYRCLE's RoB Tool 21](#_Toc4137)

[Supplementary File 6 Pairwise comparison forest plot 25](#_Toc32756)

[Supplementary File 7 Funnel plot for publication bias in all outcomes 33](#_Toc13148)

[Supplementary Table S8 Summary of subgroup analysis results 35](#_Toc25855)

# **Supplementary File 1** Database search strategy

**Pubmed**

(((((((((((((((((((((((((Animals, Laboratory[MeSH Terms]) OR (Animals, Laboratory[Title/Abstract])) OR (laboratory animal[Title/Abstract])) OR (experimental animal[Title/Abstract])) OR (animal model[Title/Abstract])) OR (preclinical model[Title/Abstract])) OR (animal subject[Title/Abstract])) OR (test animal[Title/Abstract])) OR (animal experiment[Title/Abstract])) OR (rodent[Title/Abstract])) OR (murine[Title/Abstract])) OR (mouse[Title/Abstract])) OR (mice[Title/Abstract])) OR (rat[Title/Abstract])) OR (hamster[Title/Abstract])) OR (guinea pig[Title/Abstract])) OR (rabbit[Title/Abstract])) OR (dog[Title/Abstract])) OR (Beagle[Title/Abstract])) OR (pig[Title/Abstract])) OR (non-human primate[Title/Abstract])) OR (NHP[Title/Abstract])) OR (macaque[Title/Abstract])) OR (monkey[Title/Abstract])) AND (((((((((((((((((((((((((((((((Acupuncture Therapy[MeSH Terms]) OR (Acupuncture[MeSH Terms])) OR (Electroacupuncture[MeSH Terms])) OR (Acupuncture, Ear[MeSH Terms])) OR (Moxibustion[MeSH Terms])) OR (Meridians[MeSH Terms])) OR (Acupuncture Points[MeSH Terms])) OR (Massage[MeSH Terms])) OR (Acupuncture Therapy[Title/Abstract])) OR (Acupuncture[Title/Abstract])) OR (Electroacupuncture[Title/Abstract])) OR (Acupuncture, Ear[Title/Abstract])) OR (Moxibustion[Title/Abstract])) OR (Meridians[Title/Abstract])) OR (Acupuncture Points[Title/Abstract])) OR (Massage[Title/Abstract])) OR (Needle[Title/Abstract])) OR (needling[Title/Abstract])) OR (Acupoint[Title/Abstract])) OR (Acupotomy[Title/Abstract])) OR (acupotome therapy[Title/Abstract])) OR (pricking blood[Title/Abstract])) OR (Cupping[Title/Abstract])) OR (Scraping[Title/Abstract])) OR (Guasha[Title/Abstract])) OR (hot medicinal pack[Title/Abstract])) OR (Tuina[Title/Abstract])) OR (Massotherapy[Title/Abstract])) OR (Thread-embedding[Title/Abstract])) OR (catgut embedding[Title/Abstract])) OR (warm-needle[Title/Abstract]))) AND (((((((((((Fatigue Syndrome, Chronic[MeSH Terms]) OR (Fatigue Syndrome, Chronic[Title/Abstract])) OR (chronic fatigue syndrome[Title/Abstract])) OR (CFS[Title/Abstract])) OR (myalgic encephalomyelitis[Title/Abstract])) OR (ME[Title/Abstract])) OR (post-viral fatigue syndrome[Title/Abstract])) OR (systemic exertion intolerance disease[Title/Abstract])) OR (SEID[Title/Abstract])) OR (chronic fatigue[Title/Abstract] AND immune dysfunction syndrome[Title/Abstract])) OR (CFIDS[Title/Abstract]))

**Embase**

**#1** 'chronic fatigue syndrome'/exp

**#2** 'fatigue syndrome, chronic':ti,ab,kw OR 'chronic fatigue syndrome':ti,ab,kw OR 'cfs':ti,ab,kw OR 'myalgic encephalomyelitis':ti,ab,kw OR 'me':ti,ab,kw OR 'post-viral fatigue syndrome':ti,ab,kw OR 'systemic exertion intolerance disease':ti,ab,kw OR 'seid':ti,ab,kw OR 'chronic fatigue and immune dysfunction syndrome':ti,ab,kw OR 'cfids':ti,ab,kw

**#3** #1 OR #2

**#4** 'acupuncture'/exp OR 'electroacupuncture'/exp OR 'auricular acupuncture'/exp OR 'moxibustion'/exp OR 'acupuncture point'/exp OR 'massage'/exp

**#5** 'acupuncture therapy':ti,ab,kw OR 'acupuncture':ti,ab,kw OR 'electroacupuncture':ti,ab,kw OR 'acupuncture, ear':ti,ab,kw OR 'moxibustion':ti,ab,kw OR 'meridians':ti,ab,kw OR 'acupuncture points':ti,ab,kw OR 'massage':ti,ab,kw OR 'needle':ti,ab,kw OR 'needling':ti,ab,kw OR 'acupoint':ti,ab,kw OR 'acupotomy':ti,ab,kw OR 'acupotome therapy':ti,ab,kw OR 'pricking blood':ti,ab,kw OR 'cupping':ti,ab,kw OR 'scraping':ti,ab,kw OR 'guasha':ti,ab,kw OR 'hot medicinal pack':ti,ab,kw OR 'tuina':ti,ab,kw OR 'massotherapy':ti,ab,kw OR 'thread-embedding':ti,ab,kw OR 'catgut embedding':ti,ab,kw OR 'warm-needle':ti,ab,kw

**#6** #4 OR #5

**#7** 'experimental animal'/exp

**#8** 'animals, laboratory':ti,ab,kw OR 'laboratory animal':ti,ab,kw OR 'experimental animal':ti,ab,kw OR 'animal model':ti,ab,kw OR 'preclinical model':ti,ab,kw OR 'animal subject':ti,ab,kw OR 'test animal':ti,ab,kw OR 'animal experiment':ti,ab,kw OR 'rodent':ti,ab,kw OR 'murine':ti,ab,kw OR 'mouse':ti,ab,kw OR 'mice':ti,ab,kw OR 'rat':ti,ab,kw OR 'hamster':ti,ab,kw OR 'guinea pig':ti,ab,kw OR 'rabbit':ti,ab,kw OR 'dog':ti,ab,kw OR 'beagle':ti,ab,kw OR 'pig':ti,ab,kw OR 'non-human primate':ti,ab,kw OR 'nhp':ti,ab,kw OR 'macaque':ti,ab,kw OR 'monkey':ti,ab,kw

**#9** #7 OR #8

**#10** #3 AND #6 AND #9

**Cochrane library**

**#1** MeSH descriptor: [Fatigue Syndrome, Chronic] explode all trees

**#2** ('Fatigue Syndrome, Chronic' OR 'chronic fatigue syndrome' OR 'CFS' OR 'myalgic encephalomyelitis' OR 'ME' OR 'post-viral fatigue syndrome' OR 'systemic exertion intolerance disease ' OR 'SEID' OR 'chronic fatigue and immune dysfunction syndrome' OR 'CFIDS'):ti,ab,kw

**#3** MeSH descriptor: [Acupuncture Therapy] explode all trees

**#4** MeSH descriptor: [Acupuncture] explode all trees

**#5** MeSH descriptor: [Electroacupuncture] explode all trees

**#6** MeSH descriptor: [Acupuncture, Ear] explode all trees

**#7** MeSH descriptor: [Moxibustion] explode all trees

**#8** MeSH descriptor: [Meridians] explode all trees

**#9** MeSH descriptor: [Acupuncture Points] explode all trees

**#10** MeSH descriptor: [Massage] explode all trees

**#11** ('Acupuncture Therapy' OR 'Acupuncture' OR 'Electroacupuncture' OR 'Acupuncture, Ear' OR 'Moxibustion' OR 'Meridians' OR 'Acupuncture Points' OR 'Massage' OR 'Needle' OR 'needling' OR 'Acupoint' OR 'Acupotomy' OR 'acupotome therapy ' OR 'pricking blood' OR 'Cupping' OR 'Scraping' OR 'Guasha' OR 'hot medicinal pack' OR 'Tuina' OR 'Massotherapy' OR 'Thread-embedding' OR 'catgut embedding' OR 'warm-needle'):ti,ab,kw

**#12** MeSH descriptor: [Animals, Laboratory] explode all trees

**#13** ('Animals, Laboratory' OR 'laboratory animal' OR 'experimental animal' OR 'animal model' OR 'preclinical model' OR 'animal subject' OR 'test animal' OR 'animal experiment' OR 'rodent' OR 'murine' OR 'mouse' OR 'mice' OR 'rat' OR 'hamster' OR 'guinea pig' OR 'rabbit' OR 'dog' OR 'Beagle' OR 'pig' OR 'non-human primate' OR 'NHP' OR 'macaque' OR 'monkey'):ti,ab,kw

**#14** #1 OR #2

**#15** #3 OR #4 OR #5 OR #6 OR #7 OR #8 OR #9 OR #10 OR #11

**#16** #12 OR #13

**#17** #14 AND #15 AND #16

**Web of science**

**#1** TS=("Fatigue Syndrome, Chronic" OR "chronic fatigue syndrome" OR "CFS" OR "myalgic encephalomyelitis" OR "ME" OR "post-viral fatigue syndrome" OR "systemic exertion intolerance disease " OR "SEID" OR "chronic fatigue and immune dysfunction syndrome" OR "CFIDS")

**#2** TS=("Acupuncture Therapy" OR "Acupuncture" OR "Electroacupuncture" OR "Acupuncture, Ear" OR "Moxibustion" OR "Meridians" OR "Acupuncture Points" OR "Massage" OR "Needle" OR "needling" OR "Acupoint" OR "Acupotomy" OR "acupotome therapy " OR "pricking blood" OR "Cupping" OR "Scraping" OR "Guasha" OR "hot medicinal pack" OR "Tuina" OR "Massotherapy" OR "Thread-embedding" OR "catgut embedding" OR "warm-needle")

**#3** TS=("Animals, Laboratory" OR "laboratory animal" OR "experimental animal" OR "animal model" OR "preclinical model" OR "animal subject" OR "test animal" OR "animal experiment" OR "rodent" OR "murine" OR "mouse" OR "mice" OR "rat" OR "hamster" OR "guinea pig" OR "rabbit" OR "dog" OR "Beagle" OR "pig" OR "non-human primate" OR "NHP" OR "macaque" OR "monkey")

**#4** #1 AND #2 AND #3

Note: The search strategy employed CFS-related terminology (including “chronic fatigue syndrome”, “CFS”, “myalgic encephalomyelitis” etc.) to ensure comprehensive retrieval of relevant animal studies. However, the included studies were subsequently classified as "fatigue-like condition animal models" rather than true CFS models, acknowledging the significant differences between these induced models and human CFS pathophysiology.

# **Supplementary File 2** Conversion formula for standard deviation (SD).

**Standard Error to Standard Deviation:**

SD=SE×$\sqrt{N}$ where SE is the standard error and N is the sample size.

**95% Confidence Interval to Standard Deviation:**

**1** If the sample size of the test and control groups is greater than or equal to 100: SD=$\sqrt{N}$×(Upper limit of credible intervals - Lower limit of credible intervals)/3.92

**2** If the sample size of the test and control groups is less than or equal to 60: SD=$\sqrt{N}$×(Upper limit of credible intervals - Lower limit of credible intervals)/ tinv (1-0.95,n-1)

**3** For studies with sample sizes between 60 and 100 in each group, both of the above methods can be used.

**Range** **converted SD:**

SD= (Upper limit - lower limit)/4

**Quartile** **converted SD:**

SD= (Upper limit - lower limit)/1.35

Tinv: Represent probabilities, degrees of freedom in excel sheet

# **Supplementary Table S3** Definition of interventions and controls.

| Intervention | Abbreviation | Definition |
| --- | --- | --- |
| Control group | C | The control group refers to the animal population observed under conditions where no experimental treatment is administered, or only standard basic intervention is provided. |
| Manual acupuncture | MA | Manual acupuncture is a core technique within acupuncture therapy, involving the insertion of fine needles into specific points on the body. It is guided by the principles of Traditional Chinese Medicine, aiming to regulate qi and blood, balance yin and yang, and restore health by stimulating the body’s meridians and acupoints. |
| Electroacupuncture | EA | Electroacupuncture is a therapeutic technique in which, after the insertion of needles into acupoints and the attainment of deqi (needle sensation), a microcurrent wave is applied to the needles. The current is generally administered in two main waveform modes:continuous wave and intermittent wave. This method enhances stimulation of the acupoints. |
| Fire-acupuncture | FA | Fire-acupuncture is a therapeutic technique in which the tip of a needle is heated until red-hot and then rapidly inserted into specific acupoints to treat diseases. |
| Moxibustion | MB | Moxibustion, also known as moxa therapy or moxibustion therapy, is a therapeutic method in which ignited moxa (made from dried mugwort leaves) in the form of moxa sticks or cones is used to apply heat stimulation to specific acupoints or regions of the body. By activating the flow of meridian qi, it helps regulate disordered physiological and biochemical functions, thereby achieving the purpose of preventing and treating diseases. |
| Warm needling therapy | WN | Warm needling therapy is a therapeutic technique that combines acupuncture with moxibustion. Also known as needle-handle moxibustion, it involves wrapping and igniting a small roll of moxa wool around the handle of an inserted needle after deqi is achieved and the needle is retained. The heat is then conducted through the needle body into the acupoint. Typically, one to three moxa rolls, each approximately the size of a jujube pit, are burned per treatment session. |
| Tuina | TN | Tuina is a manual therapy in which practitioners apply their hands to the patient's body surface, injured areas, sites of discomfort, specific acupoints, or painful regions. Using a variety of techniques such as pushing, grasping, pressing, rubbing, kneading, pinching, digital pressure, and tapping with appropriate force, the therapy aims to unblock meridians, promote qi and blood circulation, relieve pain, expel pathogenic factors, strengthen the body's resistance, and restore the balance between yin and yang. |
| Guasha | GS | Guasha is a therapeutic technique guided by the theory of meridians and acupoints in Traditional Chinese Medicine. It involves the use of a specially designed scraping tool, along with a corresponding scraping technique and a suitable medium, to repeatedly scrape and friction the surface of the skin. This process induces local skin reactions such as reddish millet-like spots or dark red petechiae, known as “sha” manifestation, thereby promoting blood circulation and facilitating the outward expression of pathogenic factors. |
| Thread-embedding therapy | TE | Thread-embedding therapy primarily involves the implantation of absorbable suture material into specific acupoints to achieve continuous stimulation of the body. |
| Needle-pricking | NP | Needle-pricking therapy, also known as pricking and lifting therapy or pricking-based therapeutic method, is an external treatment technique in Traditional Chinese Medicine that falls under the category of bloodletting therapy. In this method, specialized needles such as three-edged needles or sharp-tip needles are inserted into specific acupoints or reactive sites in the superficial layers of the skin, followed by the lifting and severing of subcutaneous fibrous tissues or the release of a small amount of blood. |

# **Supplementary Table S4** Basic characteristics of the included literatures

| ID | Animal models | | | | | | Intervention (T) | | | Intervention (C) | Specimen | Region | Blinding | Randomization |
| --- | --- | --- | --- | --- | --- | --- | --- | --- | --- | --- | --- | --- | --- | --- |
|  | Species | Sex | Age | Weight (g) | n  T/C | Model | Type | Duration | Acupoints |  |  |  |  |  |
| QianGF2018 | SD rats | ♂ | 6-8 weeks | 250-280 | 10/10 | Restriction + cold water swimming | FA | Once a day for a week | BL43 | Blank control | Serum | China | NR | Y |
| WangYH2018 | Wistar rats | ♂ | NR | 180-210 | 30/30 | Corticosteroid application + cold water swimming | TN | Once a day for 15 days | GV20, CV4, BL23,  KI3, SP6 | Blank control | Serum | China | NR | Y |
| HuaJS2017a | SD rats | ♂ | NR | 160-200 | 10/10 | Circadian reversal + restriction + cold water swimming | EA | 20 min, once a day for 20 days | GV20, ST36, SP6, LR3 | Blank control | Serum | China | NR | Y |
| HuaJS2017b | SD rats | ♂ | NR | 160-200 | 10/10 | Circadian reversal + restriction + cold water swimming | EA | 20 min, once a day for 20 days | GV20, ST36, SP6, LR3 | Blank control | Serum | China | NR | Y |
| ZhaiH2016 | KM mice | ♂ | 4-6 weeks | 18-22 | 12/12/12/12 | Restriction + exhaustive swimming | MA | 10 min, once a day for 7 days | T1: LI4, LR3  T2: LI4, LR3, SP6  T3: LI4, LR3, ST36 | Blank control | Serum | China | NR | Y |
| ZhaoXM2013 | Wistar rats | ♂:♀1:1 | NR | 180-220 | 10/10/10 | Treadmill Exhaustive Exercise + intraperitoneal injection of p-chlorophenylalanine | MA | 10 min, once a day for 10 days | T1: BL23, HT7, GV20, ST36  T2: MA + Suanzaoren Decoction | Diazepam, 0.83 mg/kg, once a day for 10 days | Brain, blood | China | NR | Y |
| ChengZD2013a | SD rats | ♀ | NR | 160-180 | 15/15 | Exhaustive swimming + restriction | MB | 15 min, once a day for 21 days | CV4 | Blank control | Serum | China | NR | Y |
| ChengZD2013b | SD rats | ♂:♀1:1 | NR | 180-220 | 10/10 | Dietary inadequacy + cold water swimming | MB | 10 min, once a day for 21 days | CV8 | Blank control | Feces | China | NR | Y |
| ChengZD2013c | SD rats | ♀ | NR | 180-220 | 10/10 | Dietary inadequacy + cold water swimming | MB | 10 min, once a day for 21 days | CV4 | Blank control | Feces | China | NR | Y |
| LuoWJ2012 | SD rats | ♂:♀1:1 | NR | 160-180 | 10/10/10 | Restriction + cold water swimming | T1: MA  T2: MB | T1: 20 min, once a day for 14 days  T2: 10 min, once a day for 14 days | BL13, BL15, BL18, BL20, BL23 | Blank control | Serum, spleen | China | NR | Y |
| ZhouP2018a | SD rats | ♂ | NR | 170-190 | 15/15 | Restriction + sleep deprivation + load  swimming | EA | 20 min, once a day for 20 days | BL13, BL15, BL18, BL20, BL23 | Blank control | Brain, serum | China | NR | Y |
| ZhouP2018b | SD rats | ♂ | NR | 170-190 | 15/15 | Restriction + sleep deprivation + load  swimming | EA | 20 min, once a day for 20 days | BL13, BL15, BL18, BL20, BL23 | Blank control | Serum | China | NR | Y |
| WangDL2018 | SD rats | ♂ | NR | 190-210 | 12/12 | Exhausted swimming + sleep deprivation + dietary inadequacy | EA | 30 min, once a day for 15 days | GV20, frontal lobe area, parietal lobe area | Blank control | Brain | China | NR | Y |
| LinYM2017 | SD rats | ♂ | NR | 160-200 | 15/15 | Sleep deprivation + exhaustive swimming | MB | 10 min, once a day for 15 days | CV4, CV6 | Blank control | Serum | China | NR | Y |
| YangTS2016 | SD rats | ♂ | NR | 190-210 | 12/12 | Exhaustive swimming | EA | 30 min, once a day for 15 days | GV20, Emotional Line 1, Sensory Area | Blank control | Brain | China | NR | Y |
| SunH2014 | SD rats | ♂ | NR | 180-220 | 10/10 | Exhaustive swimming + sleep deprivation | MB | 8 min, once a day for 14 days | ST36, BL13 | Blank control | Serum | China | NR | Y |
| YangTS2014 | SD rats | ♂ | NR | 190-210 | 12/12 | Exhaustive swimming + dietary inadequacy + circadian reversal | EA | 30 min, once a day for 15 days | GV20, Emotional Line 1, Sensory Area | Blank control | Serum | China | NR | Y |
| ZhaoL2014 | Wistar rats | NR | NR | 160-220 | 18/17 | Exhaustive swimming + sleep deprivation | MB | 8 min, once a day for 15 days | BL23, ST36 | Blank control | Blood | China | NR | Y |
| MaoX2013 | Wistar rats | ♂ | NR | 180-220 | 15/15 | Restriction + circadian reversal+ exhaustive swimming | MB | 15 min, once a day for 21 days | GV20, BL18, BL20, BL23, ST36 | Blank control | Brain | China | NR | Y |
| ZengJ2013a | SD rats | ♂ | 3-4 months | 180-220 | 12/12 | Suspension + cold water swimming | GS | once every 2 days for 12 days | GV, BL, CV | Blank control | Serum, thymus, spleen | China | NR | Y |
| ChengCS2013 | SD rats | ♂:♀1:1 | NR | 181.06-202.46 | 12/12 | Cold water swimming + restriction | T1: EA+CRF  T2:EA+CRFA | 20 min, once a day for 3 days | BL23, ST36 | C1: CRF  C2: CRFA | Plasma, brain | China | NR | Y |
| LvHZ2012 | SD rats | ♂ | 3-4 months | 180-220 | 9/9/9/9 | Suspension + cold water swimming | T1:GS  T2:MB  T3:TE | T1:once every 2 days for 6 days  T2:1 min, once a day for 12 days  T3:12 days | TI:GV, BL, CV  T2:BL18, BL20, BL23  T3:BL18, BL20, BL23 | Blank control | NR | China | NR | Y |
| ChengCS2012 | SD rats | ♂:♀1:1 | NR | 181.06-202.46 | 12/12 | Suspension + cold water swimming | T1: EA+CRF  T2:EA+CRFA | 20 min, once a day for 3 days | BL23, ST36 | C1: CRF  C2: CRFA | NR | China | NR | Y |
| LuoYH2012a | SD rats | ♂:♀1:1 | 3 months | 160-180 | 10/10 | Suspension + cold water swimming | MB | 10 min, once a day for 14 days | BL13, BL15, BL18, BL20, BL23 | Blank control | Serum | China | NR | Y |
| YaoF2011 | SD rats | ♂ | NR | 270-290 | 10/10 | Sleep deprivation + exhaustive swimming | TN | 5 min, once a day for 28 days | Back-shu points | Blank control | NR | China | NR | Y |
| ZengJ2011 | SD rats | ♂ | 3-4 months | 180-220 | 12/12 | Suspension + cold water swimming | MB | once every 2 days for 12 days | BL18, BL20, BL23 | Blank control | Serum, thymus, spleen | China | NR | Y |
| ChenP2010 | SD rats | ♂ | NR | 180-220 | 10/10 | Restriction | MB | 60 min, once a day for 14 days | BL13, BL15, BL18, BL20, BL23 | Blank control | NR | China | NR | Y |
| LvHZ2009a | SD rats | ♂ | 3-4 months | 180-220 | 9/9 | Suspension + cold water swimming | MB | 1 min, once a day for 12 days | BL18, BL20, BL23 | Blank control | Brain. plasma | China | NR | Y |
| ChenXH2009a | SD rats | ♂ | 3 months | 180-220 | 10/10 | Restriction | MB | 20 min, once a day for 14 days | BL13, BL15, BL18, BL20, BL23 | Blank control | NR | China | NR | Y |
| ChenXH2009b | SD rats | ♂ | 3 months | 180-220 | 10/10 | Restriction | MB | 20 min, once a day for 14 days | BL13, BL15, BL18, BL20, BL23 | Blank control | Brain | China | NR | Y |
| ChenYF2007a | Wistar rats | ♂ | NR | 160-180 | 11/11 | Cold water swimming | EA | 20 min, once a day for 9 days | GV20, ST36 | Blank control | Brain, serum | China | NR | Y |
| ChenYF2007b | Wistar rats | ♂ | NR | 160-180 | 11/11 | Cold water swimming | EA | 20 min, once a day for 9 days | GV20, ST36 | Blank control | Brain, plasma | China | NR | Y |
| MaYH2006 | Wistar rats | ♂ | NR | 160-180 | 8/8 | Cold water swimming + corticosteroid application | EA | 30 min, once a day for 21 days | GV20, ST36, KI3 | Blank control | Spleen | China | NR | Y |
| WuSJ2006 | Wistar rats | ♂ | NR | 160-180 | 12/12 | Cold water swimming + corticosteroid application | EA | 30 min, once a day for 21 days | GV20, KI3 | Blank control | Brain | China | NR | Y |
| WangJJ2004 | Wistar rats | ♂ | NR | 160-190 | 8/8 | Restriction + cold water swimming | EA | 20 min, once a day for 21 days | T1:LI4, LR3  T2:LR3, GV20, ST36 | Blank control | NR | China | NR | Y |
| MengH2003a | Wistar rats | ♂ | NR | 160-180 | 10/10 | Restriction + cold water swimming | EA | 20 min, once a day for 21 days | LI4, LR3 | Blank control | Serum | China | NR | Y |
| MengH2003b | Wistar rats | ♂ | NR | 160-180 | 8/8 | Restriction + cold water swimming | EA | 30 min, once a day for 21 days | LR3, GV20, ST36 | Blank control | Brain | China | NR | Y |
| LiuP2018 | SD rats | ♂:♀  1:1 | NR | 184.63-215.87 | 18/18 | Restriction + cold water swimming | WN | 30 min, once a day for 5 days | BL23, ST36 | Blank control | Serum | China | NR | Y |
| MinXL2012 | SD rats | ♀ | NR | 150-250 | 10/10 | Readmill exhaustive exercise + intraperitoneal injection of p-chlorophenylalanine | MA+TE | 20 min, once a day | GV20 | Blank control | Brain | China | NR | Y |
| ZengJ2013b | SD rats | ♂ | 3-4 months | 180-220 | 12/12 | Suspension + cold water swimming | GS | once every 2 days for 12 days | GV, BL, CV | Blank control | NR | China | NR | Y |
| ZhuMM2010 | SD rats | ♂:♀  1:1 | 3 months | 160-180 | 12/12/12 | Restriction + cold water swimming | T1:MA  T2:MB | T1:30 min  T2:10 min | BL13, BL15, BL18, BL20, BL23 | Blank control | NR | China | NR | Y |
| QianGF2019 | SD rats | ♂ | 6-8 weeks | 220-280 | 10/10 | Restriction + cold water swimming | NP | once every 2 days for 7 days | BL43 | Blank control | Serum | China | NR | Y |
| ZhaiCT2025a | SD rats | ♂:♀  1:1 | 6-8 weeks | 180-220 | 10/10 | Restriction + exhaustive swimming | MB | 15 min, once a day for 10 days | CV8, CV4, ST36, GB14 | Blank control | Serum | China | NR | Y |
| LiHY2025 | SD rats | ♂ | NR | 190-230 | 8/8 | Exhaustive swimming + restriction + dietary inadequacy | WN | 15 min, once a day for 14 days | CV4, CV12, ST36 | Blank control | NR | China | Y | Y |
| ChenYF2007c | Wistar rats | ♂ | NR | 160-180 | 11/11 | Cold water swimming | EA | 20 min, once a day for 9 days | GV20, ST36 | Blank control | Brain | China | NR | Y |
| ZhangWJ2015 | SD rats | ♂ | 6-8 weeks | 180-220 | 8/8/8 | Restriction + cold water swimming | T1:EA  T2:EA+TCM | 20 min, once a day for 7 days | BL20 | Blank control | Serum | China | NR | Y |
| LiuCZ2021 | SD rats | ♂ | NR | 180-220 | 12/12 | Suspension + cold water swimming | MA | 20 min, once a day for 12 days | GV20, ST36, SP6 | Blank control | Brain | China | NR | Y |
| FengYY2023 | SD rats | ♂ | NR | 150-180 | 6/6 | Restriction | MA | 20 min, once a day for 10 days | BL13, BL20, BL23 | Blank control | Brain | China | NR | Y |
| TangCZ2007 | SD rats | ♂ | NR | 190-220 | 10/10 | Restriction + cold water swimming | MA | 30 min, once a day for 30 days | GV20, ST36, BL23 | Blank control | Serum | China | NR | Y |
| LuoYH2006 | SD rats | ♂ | NR | 160-180 | 10/10 | Restriction + cold water swimming | MA | 20 min, once a day for 14 days | GV20, ST36, BL23 | Blank control | Serum | China | NR | Y |
| ZengYH2009 | SD rats | ♂ | NR | 190-220 | 10/10 | Restriction + cold water swimming | MA | 20 min, once a day for 14 days | GV20, ST36, BL23 | Blank control | NR | China | NR | Y |
| QuCJ2010 | SD rats | ♀ | NR | 180-260 | 15/15 | Restriction + cold water swimming + circadian reversal | EA | 15 min, once a day for 21 days | GV20, ST36, LR3 | Blank control | Serum, brain | China | NR | Y |
| LuoYH2012b | SD rats | ♂:♀  1:1 | 3 months | 160-180 | 10/10 | Restriction + cold water swimming | MA | 20 min, once a day for 14 days | BL13,  BL15,  BL18, BL20,  BL23 | Blank control | Serum | China | NR | Y |
| ZouJ2010 | SD rats | ♂ | 2 months | 196-247 | 12/12 | Restriction + cold water swimming + electric shock | MB | 15-20 min, once a day | ST36 | Blank control | Blood | China | NR | Y |
| LvHZ2009b | SD rats | ♂ | 3-4 months | 180-220 | 9/9 | Suspension + cold water swimming | TE | 12 days | BL18, BL20,  BL23 | Blank control | Brain, plasma | China | NR | Y |
| LuYX2024 | Wistar rats | ♂ | 6-8 weeks | 180-220 | 7/7 | Sleep deprivation + restriction + cold water swimming | MB | 5 min, once a day for 28 days | CV8 | Blank control | Serum | China | NR | Y |
| ShuiL2021a | SD rats | ♂ | NR | 160-180 | 10/10 | exhaustive swimming | WN | 15 min, once a day for 11 days | Dinghui acu  point and Xin acupoin | Blank control | Serum, brain | China | NR | Y |
| SiQ2020 | SD rats | ♂ | NR | 160-180 | 8/8 | exhaustive swimming | WN | 15 min, once a day for 11 days | Dinghui acu  point and Xin acupoin | Blank control | Brain | China | NR | Y |
| YiRN2020 | SD rats | ♂ | NR | 110-130 | 10/10 | exhaustive swimming | WN | 15 min, once a day for 11 days | Dinghui acu  point and Xin acupoin | Blank control | Serum | China | NR | Y |
| ShuiL2021b | SD rats | ♂ | NR | 160-180 | 10/10 | exhaustive swimming | WN | 15 min, once a day for 11 days | Dinghui acu  point and Xin acupoin | Blank control | Brain | China | NR | Y |
| YangY2025 | SD rats | ♂ | 5-6 weeks | 180-220 | 12/12 | exhaustive exercise + restriction + sleep deprivation | EA | 15 min, once a day for 28 days | GV14, GV20 | Blank control | Feces | China | NR | Y |
| HanL2023 | SD rats | ♂ | NR | 200-220 | 12/12 | Exhaustive swimming + dietary inadequacy + sleep deprivation | EA | 30 min, once a day for 14 days | GV20, bilateral sensory area,  Ningshen | Blank control | Plasma | China | NR | Y |
| ZhaiCT2025b | SD rats | ♂:♀  1:1 | 6-8 weeks | 190-220 | 10/10 | Restriction + exhaustive swimming | MB | 5 moxa cones per point, once a day for 10 days | CV8,  CV4, ST36, LR14 | Blank control | Serum | China | NR | Y |
| LiuCZ2022 | SD rats | ♂ | NR | 180-220 | 12/12 | Suspension + cold water swimming | MB | 10 min, once a day for 15 days | CV8 | Blank control | Serum | China | NR | NR |
| YueY2022 | Wistar rats | ♂ | 3 months | 140-180 | 10/10 | Restriction + cold water swimming | TN | 30 min, once a day for 14 days | BL43 | Jin-Gui-Shen-Qi-Wan | Serum | China | NR | Y |
| YangXH2025 | SD rats | ♂ | NR | 200-250 | 8/8 | Cold water swimming | TN | 20 min, once a day for 14 days | BL | Blank control | Muscle | China | NR | Y |
| LiuCZ2024 | SD rats | ♂ | NR | 180-220 | 8/8 | Suspension+cold water swimming | MB/MA/EA | 20 min, once a day for 18 days | ST36, GV20, SP6 | Blank control | Serum | China | NR | Y |
| YangY2018 | SD rats | ♂ | NR | 180-220 | 12/12 | cold water swimming | EA | 30 min, once a day for 15 days | GV20, bilateral sensory area,  Ningshen | Blank control | Brain | China | NR | Y |
| YangY2019 | SD rats | ♂ | NR | 200-240 | 12/12 | cold water swimming | EA | 30 min, once a day for 14 days | GV20, bilateral sensory area,  Ningshen | Blank control | Serum | China | NR | Y |
| YangTS2018 | SD rats | ♂ | NR | 190-250 | 12/12 | cold water swimming | EA | 30 min, once a day for 15 days | GV20, bilateral sensory area,  Ningshen | Blank control | Brain | China | NR | NR |
| SuZC2024 | SD rats | ♂ | 6-8 weeks | 160-200 | 20/20 | sleep deprivation + cold water swimming | TN | 30 min, once a day for 42 days | Abdomen | Blank control | Serum | China | NR | Y |
| LiHN2019a | SD rats | ♂ | NR | 200-250 | 10/10 | Suspension + cold water swimming | TN | 12 min, once a day for 14 days | Abdomen | Blank control | Brain | China | NR | Y |
| LiHN2019b | SD rats | ♂ | NR | 200-250 | 10/10 | Suspension + cold water swimming | TN | 6 min, once a day for 14 days | Abdomen | Blank control | Brain | China | NR | Y |
| AnCF2024 | SD rats | ♂ | 4-5 weeks | 220-250 | 10/10 | Restriction + cold water swimming | TN | 12 min, once a day for 14 days | CV4, CV12 | Blank control | Brain | China | NR | Y |
| PanMZ2021 | Wistar rats | ♂ | NR | 190-260 | 20/20 | Suspension + cold water swimming | TN | 6 min, once a day for 14 days | CV4, CV12 | Blank control | Serum | China | NR | Y |
| XuXS2023 | SD rats | ♂:♀  1:1 | NR | 180-220 | 10/10 | Restriction + cold water swimming | MB | once a day for 10 days | CV4, CV8, ST6, LR14 | Blank control | Serum, liver, spleen, muscle | China | NR | Y |
| XuXS2025 | SD rats | ♂:♀1:1 |  | 180-220 | 10/10 | Restriction + exhaustive swimming | MB | once a day for 10 days | CV8,  CV4,  ST36,  LR14 | Blank control | Serum | China | NR | Y |

Note: T: treatment Group; C: control group; MA: manual acupuncture; EA: electroacupuncture; FA: fire-acupuncture; MB: moxibustion; WN: warm needling therapy; TN: tuina; GS: guasha; TE: thread-embedding therapy; NP: needle-pricking.

# **Supplementary Table S5** Results of risk of bias assessment using SYRCLE's RoB Tool

| ID | Selection bias | | | Performance bias | | Detection bias | | Attrition bias | Reporting bias | Other |
| --- | --- | --- | --- | --- | --- | --- | --- | --- | --- | --- |
|  | Sequence generation | Baseline characteristics | Allocation concealment | Random housing | Blinding | Random outcome assessment | Blinding | Incomplete outcome data | Selective outcome reporting | Other sources of bias |
| QianGF2018 | L | L | UN | UN | H | UN | H | L | L | UN |
| WangYH2018 | UN | UN | UN | UN | UN | UN | UN | H | L | UN |
| HuaJS2017a | UN | L | UN | UN | UN | H | UN | H | L | UN |
| HuaJS2017b | UN | L | UN | UN | UN | H | UN | H | L | UN |
| ZhaiH2016 | UN | UN | UN | UN | H | H | H | L | UN | UN |
| ZhaoXM2013 | UN | UN | UN | UN | UN | UN | UN | L | L | UN |
| ChengZD2013a | L | UN | UN | UN | H | UN | UN | UN | L | UN |
| ChengZD2013b | UN | UN | UN | H | H | UN | UN | L | L | UN |
| ChengZD2013c | UN | UN | UN | H | H | UN | UN | L | L | UN |
| LuoWJ2012 | UN | UN | UN | UN | UN | UN | UN | UN | UN | UN |
| ZhouP2018a | UN | L | UN | UN | UN | UN | UN | L | L | UN |
| ZhouP2018b | UN | L | UN | UN | UN | UN | UN | L | L | UN |
| WangDL2018 | UN | UN | UN | UN | UN | UN | UN | L | L | UN |
| LinYM2017 | L | L | UN | UN | UN | H | UN | H | L | UN |
| YangTS2016 | UN | UN | UN | UN | UN | UN | UN | L | L | UN |
| SunH2014 | UN | UN | UN | UN | UN | UN | UN | L | L | UN |
| YangTS2014 | UN | UN | UN | UN | UN | UN | UN | L | L | UN |
| ZhaoL2014 | L | L | UN | UN | H | H | H | L | L | UN |
| MaoX2013 | L | UN | UN | UN | UN | H | UN | UN | L | UN |
| ZengJ2013a | L | L | UN | UN | H | UN | UN | L | L | UN |
| ChengCS2013 | L | L | UN | UN | H | UN | UN | L | L | UN |
| LvHZ2012 | L | L | UN | UN | UN | H | UN | UN | L | UN |
| ChengCS2012 | L | L | UN | UN | H | UN | UN | L | L | UN |
| LuoYH2012a | UN | UN | UN | UN | UN | UN | UN | UN | UN | UN |
| YaoF2011 | L | L | UN | UN | H | UN | UN | L | L | UN |
| ZengJ2011 | L | L | UN | UN | H | UN | UN | L | L | UN |
| ChenP2010 | UN | L | UN | H | H | UN | H | L | L | UN |
| LvHZ2009a | L | L | UN | UN | UN | H | UN | UN | L | UN |
| ChenXH2009a | UN | L | UN | H | H | UN | H | L | L | UN |
| ChenXH2009b | UN | L | UN | H | H | UN | UN | L | L | UN |
| ChenYF2007a | UN | L | UN | H | H | UN | UN | L | L | UN |
| ChenYF2007b | UN | L | UN | H | H | UN | UN | L | L | UN |
| MaYH2006 | L | L | UN | UN | UN | H | UN | UN | L | UN |
| WuSJ2006 | UN | UN | UN | UN | UN | UN | UN | L | L | UN |
| WangJJ2004 | UN | UN | UN | UN | UN | UN | UN | L | L | UN |
| MengH2003a | L | UN | UN | UN | UN | H | UN | UN | L | UN |
| MengH2003b | UN | UN | UN | UN | UN | UN | UN | UN | UN | UN |
| LiuP2018 | L | L | UN | UN | UN | H | UN | H | L | UN |
| MinXL2012 | L | L | UN | UN | H | UN | H | L | L | UN |
| ZengJ2013b | UN | UN | UN | UN | H | H | H | L | UN | UN |
| ZhuMM2010 | L | UN | UN | UN | UN | UN | UN | UN | L | UN |
| QianGF2019 | L | L | UN | UN | H | UN | H | L | L | UN |
| ZhaiCT2025a | UN | L | UN | UN | H | H | H | L | L | UN |
| LiHY2025 | L | L | UN | UN | UN | H | UN | H | L | UN |
| ChenYF2007c | UN | L | UN | H | H | UN | UN | L | L | UN |
| ZhangWJ2015 | UN | UN | UN | UN | H | H | H | L | UN | UN |
| LiuCZ2021 | L | L | UN | UN | UN | H | UN | H | L | UN |
| FengYY2023 | UN | L | UN | H | H | UN | UN | L | L | UN |
| TangCZ2007 | UN | UN | UN | UN | UN | UN | UN | L | L | UN |
| LuoYH2008 | UN | UN | UN | UN | UN | UN | UN | UN | L | UN |
| ZengYH2009 | UN | UN | UN | UN | H | H | H | L | UN | UN |
| QuCJ2010 | L | L | UN | UN | H | UN | H | H | L | UN |
| LuoYH2012b | L | UN | UN | UN | UN | H | UN | UN | L | UN |
| ZouJ2010 | L | L | UN | UN | UN | UN | UN | H | L | UN |
| LvHZ2009b | L | L | UN | UN | UN | H | UN | UN | L | UN |
| LuYX2024 | L | L | UN | UN | UN | H | UN | UN | H | UN |
| ShuiL2021a | L | L | UN | UN | H | UN | H | L | L | UN |
| SiQ2020 | L | L | UN | UN | H | UN | H | L | L | UN |
| YiRN2020 | L | L | UN | UN | H | UN | UN | L | L | UN |
| ShuiL2021b | L | L | UN | UN | H | UN | H | L | L | UN |
| YangY2025 | L | L | UN | UN | H | UN | UN | L | L | UN |
| HanL2023 | UN | L | UN | H | H | UN | UN | L | L | UN |
| ZhaiCT2025b | UN | L | UN | UN | H | H | H | L | L | UN |
| LiuCZ2022 | L | L | UN | UN | UN | H | UN | H | L | UN |
| YueY2022 | L | L | UN | UN | H | UN | UN | L | L | UN |
| YangXH2025 | L | L | UN | UN | H | UN | UN | L | L | UN |
| LiuCZ2024 | L | L | UN | UN | UN | H | UN | H | L | UN |
| YangY2018 | L | L | UN | UN | H | UN | UN | L | L | UN |
| YangY2019 | L | L | UN | UN | H | UN | UN | L | L | UN |
| YangTS2018 | UN | UN | UN | UN | UN | UN | UN | L | L | UN |
| SuZC2024 | UN | UN | UN | UN | UN | UN | UN | L | L | UN |
| LiHN2019a | UN | L | UN | UN | UN | H | UN | H | L | UN |
| LiHN2019b | UN | L | UN | UN | UN | H | UN | H | L | UN |
| AnCF2024 | L | UN | UN | UN | H | UN | UN | L | L | UN |
| PanMZ2021 | L | L | UN | UN | H | UN | H | L | L | UN |
| XuXS2023 | UN | UN | UN | UN | UN | UN | UN | L | L | UN |
| XuXS2025 | L | L | UN | UN | UN | UN | UN | L | L | UN |

Note: L: low risk of bias; H: high risk of bias; UN: unclear.

# **Supplementary File 6** Pairwise comparison forest plot

**S6.1** Fatigue-related outcomes

**Figure S6.1-1** Pairwise comparison forest plot of exhaustion time in forced swim test. Note: C: control group; MA: manual acupuncture; EA: electroacupuncture; FA: fire-acupuncture; MB: moxibustion; WN: warm needling therapy; TN: tuina; GS: guasha; TE: thread-embedding therapy.


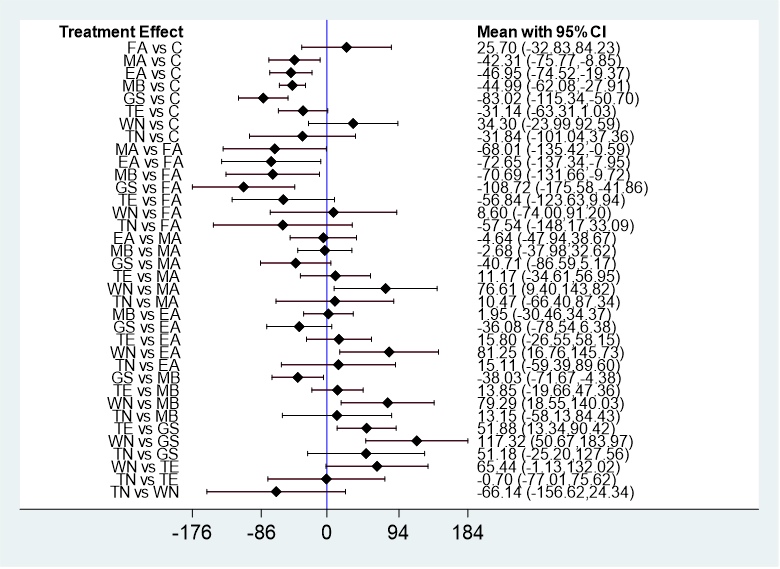


**Figure S6.1-2** Pairwise comparison forest plot of immobility time in tail suspension test. Note: C: control group; MA: manual acupuncture; EA: electroacupuncture; FA: fire-acupuncture; MB: moxibustion; WN: warm needling therapy; TN: tuina; GS: guasha; TE: thread-embedding therapy.


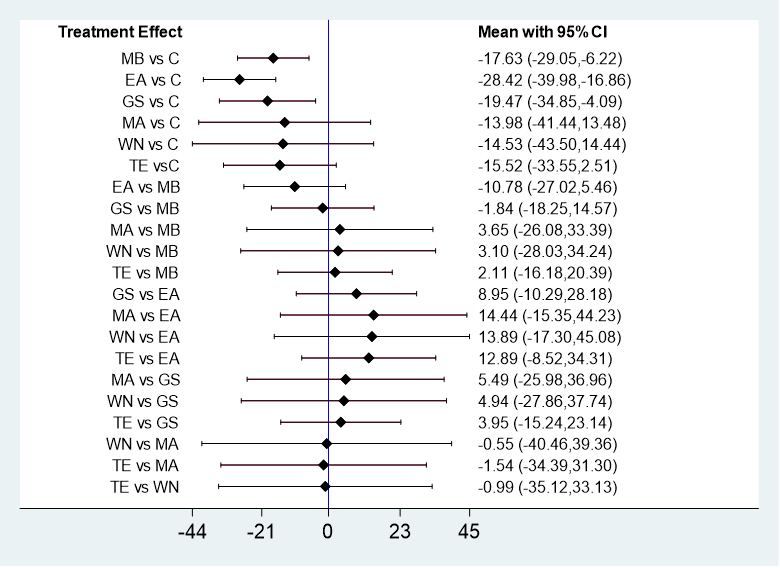
**Figure S6.1-3** Pairwise comparison forest plot of escape latency in morris water maze. Note: C: control group; MA: manual acupuncture; EA: electroacupuncture; FA: fire-acupuncture; MB: moxibustion; WN: warm needling therapy; TN: tuina; GS: guasha; TE: thread-embedding therapy.


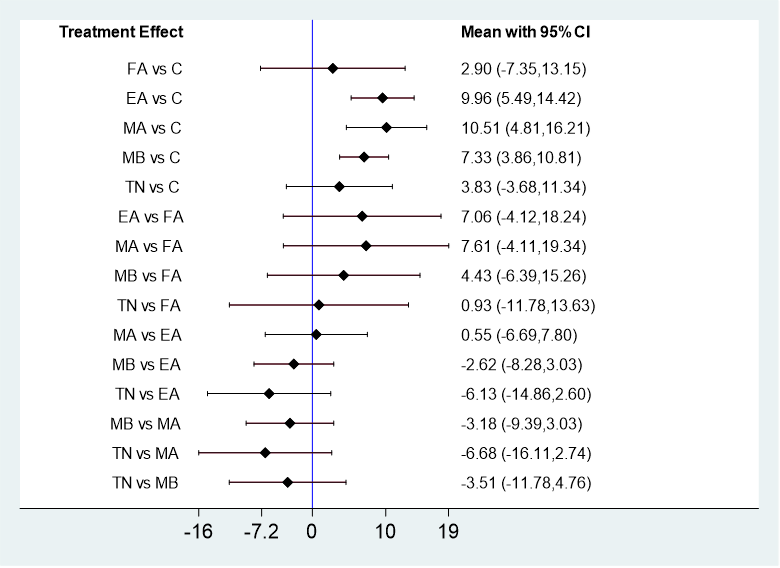
**Figure S6.1-4** Pairwise comparison forest plot of vertical score in open field test. Note: C: control group; MA: manual acupuncture; EA: electroacupuncture; FA: fire-acupuncture; MB: moxibustion; WN: warm needling therapy; TN: tuina; GS: guasha; TE: thread-embedding therapy.


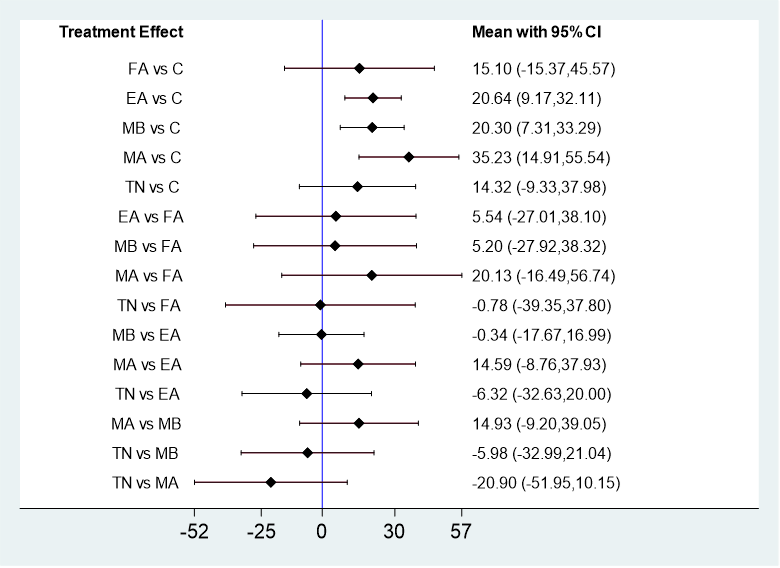
**Figure S6.1-5** Pairwise comparison forest plot of horizontal score in open field test. Note: C: control group; MA: manual acupuncture; EA: electroacupuncture; FA: fire-acupuncture; MB: moxibustion; WN: warm needling therapy; TN: tuina; GS: guasha; TE: thread-embedding therapy.


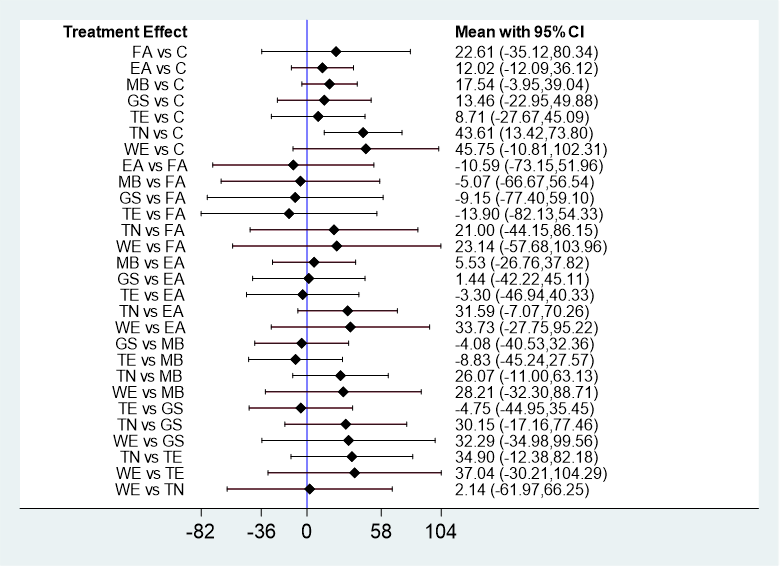
**Figure S6.1-6** Pairwise comparison forest plot of body weight. Note: C: control group; MA: manual acupuncture; EA: electroacupuncture; FA: fire-acupuncture; MB: moxibustion; WN: warm needling therapy; TN: tuina; GS: guasha; TE: thread-embedding therapy.

**S6.2** Oxidative stress-related outcomes


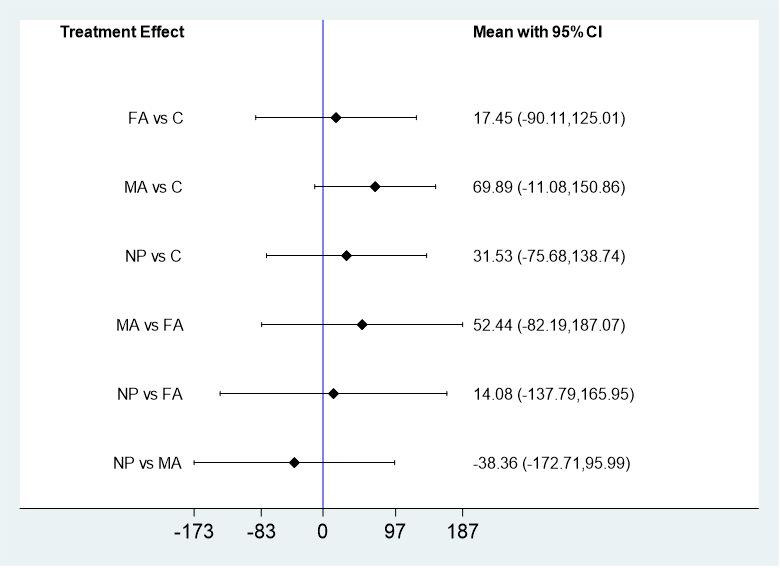
**Figure S6.2-1** Pairwise comparison forest plot of SOD level. Note: C: control group; MA: manual acupuncture; FA: fire-acupuncture; MB: moxibustion; NP: needle-pricking.


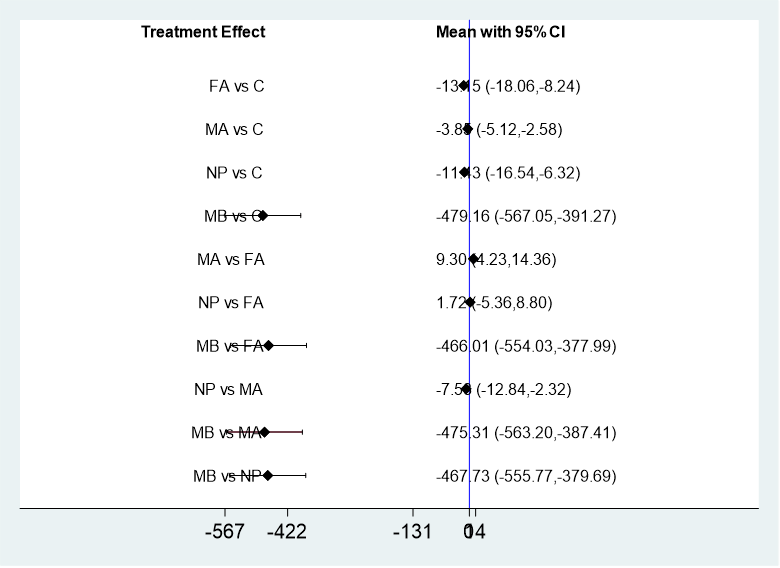
**Figure S6.2-2** Pairwise comparison forest plot of MDA level. Note: C: control group; MA: manual acupuncture; FA: fire-acupuncture; MB: moxibustion; NP: needle-pricking.

**S6.3** Inflammation-related outcomes


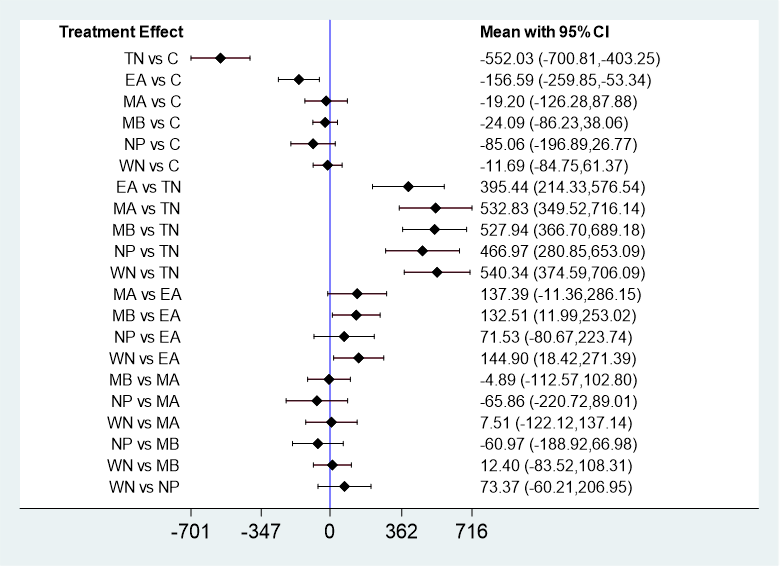
**Figure S6.3-1** Pairwise comparison forest plot of IL-1βlevel. Note: C: control group; MA: manual acupuncture; EA: electroacupuncture; MB: moxibustion; WN: warm needling therapy; NP: needle-pricking.


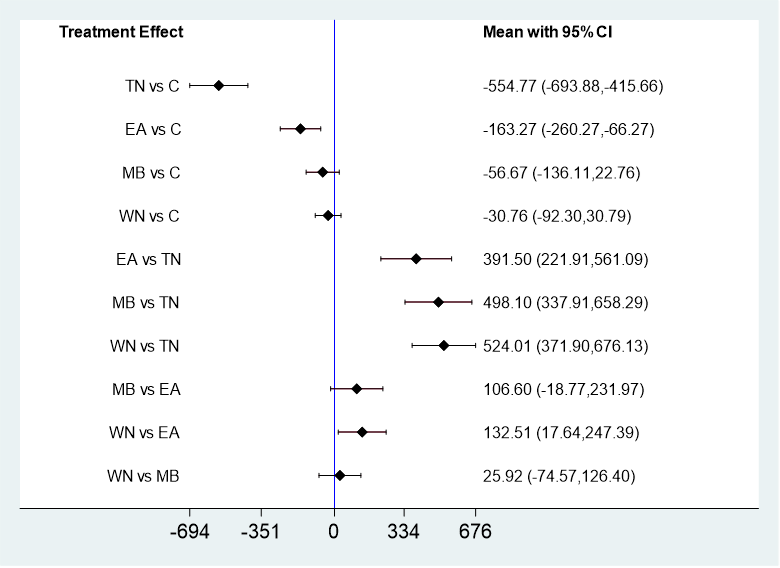
**Figure S6.3-2** Pairwise comparison forest plot of IL-6 level. Note: C: control group; MA: manual acupuncture; EA: electroacupuncture; MB: moxibustion; WN: warm needling therapy; NP: needle-pricking.


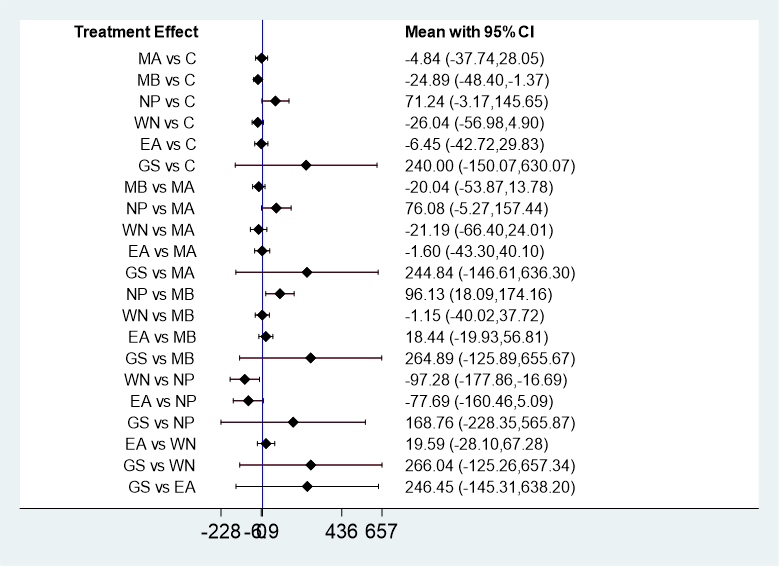
**Figure S6.3-3** Pairwise comparison forest plot of TNF-α. Note: C: control group; MA: manual acupuncture; EA: electroacupuncture; MB: moxibustion; WN: warm needling therapy; NP: needle-pricking.


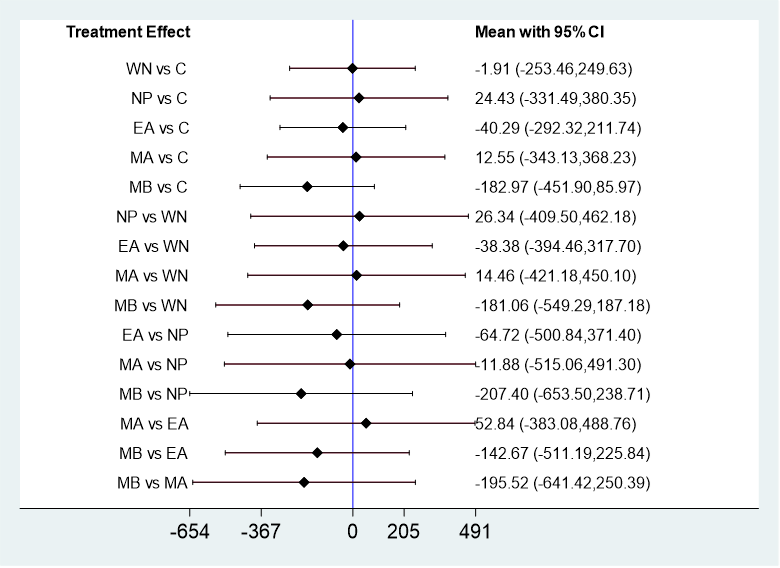
**Figure S6.3-4** Pairwise comparison forest plot of IFN-γ. Note: C: control group; MA: manual acupuncture; EA: electroacupuncture; MB: moxibustion; WN: warm needling therapy; NP: needle-pricking.

**S6.4** Endocrine-related outcomes


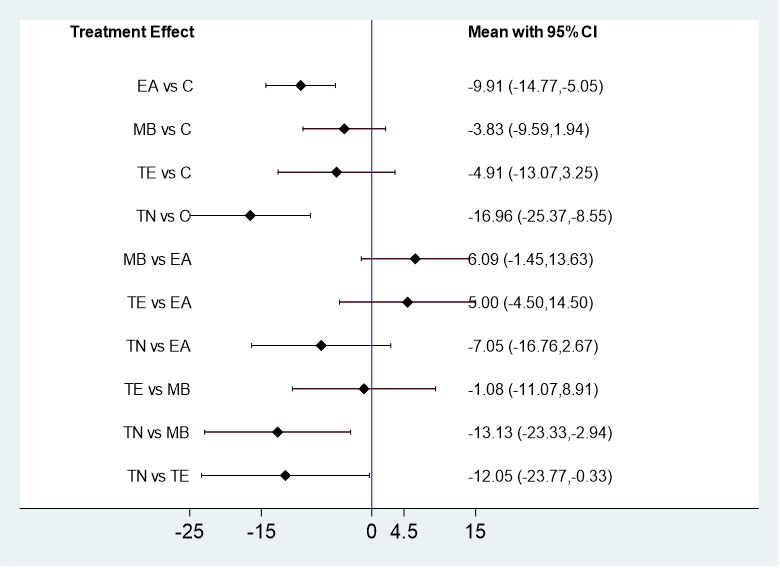
**Figure S6.4-1** Pairwise comparison forest plot of CRH level. Note: C: control group; EA: electroacupuncture; MB: moxibustion; TN: tuina; TE: thread-embedding therapy.


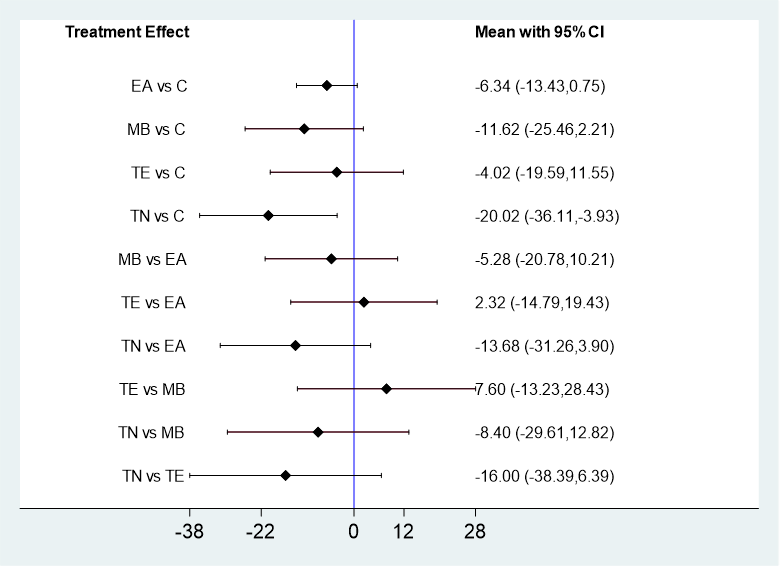
**Figure S6.4-2** Pairwise comparison forest plot of ACTH level. Note: C: control group; EA: electroacupuncture; MB: moxibustion; TN: tuina; TE: thread-embedding therapy.


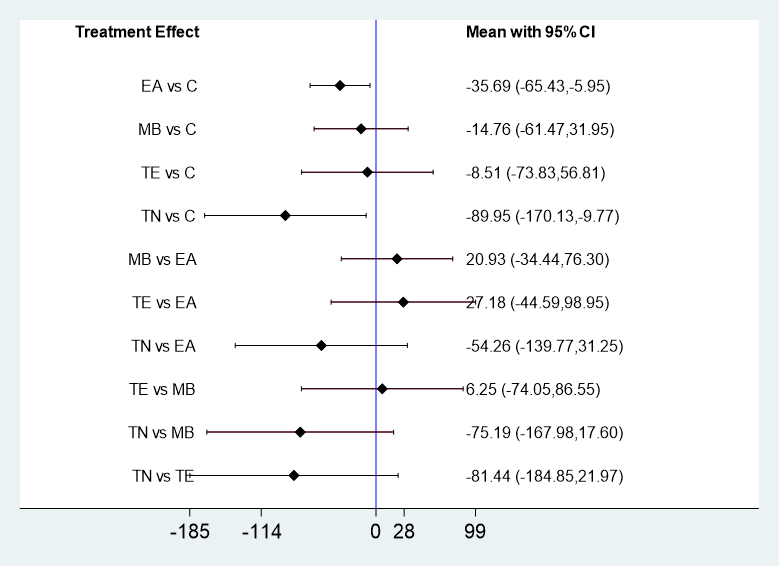
**Figure S6.4-3** Pairwise comparison forest plot of CORT level. Note: C: control group; EA: electroacupuncture; MB: moxibustion; TN: tuina; TE: thread-embedding therapy.

# **Supplementary File 7** Funnel plot for publication bias in all outcomes

**Figure S7.1** Funnel plot for publication bias in
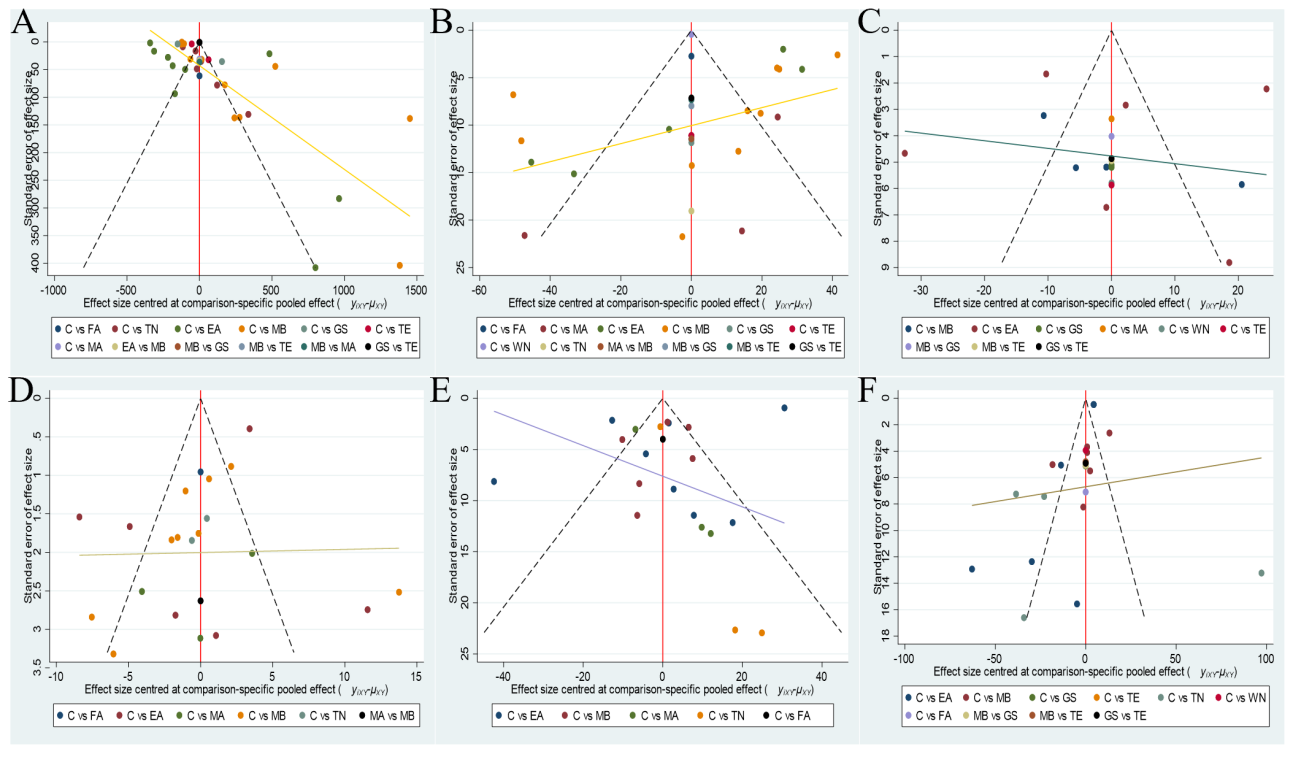
 fatigue-related outcomes. **A** Exhaustion time in forced swim test; **B** Immobility time in tail suspension test; **C** Escape latency in morris water maze; **D** Vertical score in open field test; **E** Horizontal score in open field test; **F** Body weight. Note: C: control group; MA: manual acupuncture; EA: electroacupuncture; FA: fire-acupuncture; MB: moxibustion; WN: warm needling therapy; TN: tuina; GS: guasha; TE: thread-embedding therapy.


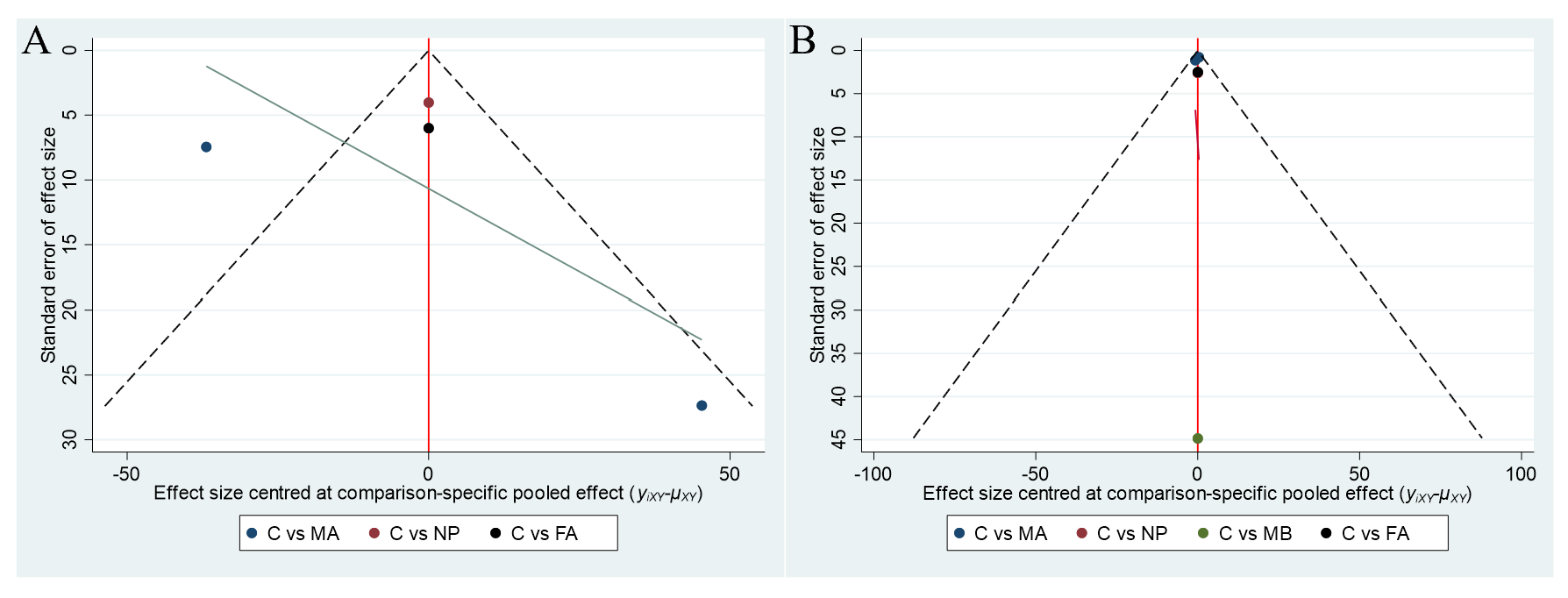
**Figure S7.2** Funnel plot for publication bias in oxidative stress-related outcomes. **A** SOD level; **B** MDA level. Note: C: control group; MA: manual acupuncture; FA: fire-acupuncture; MB: moxibustion; NP: needle-pricking.


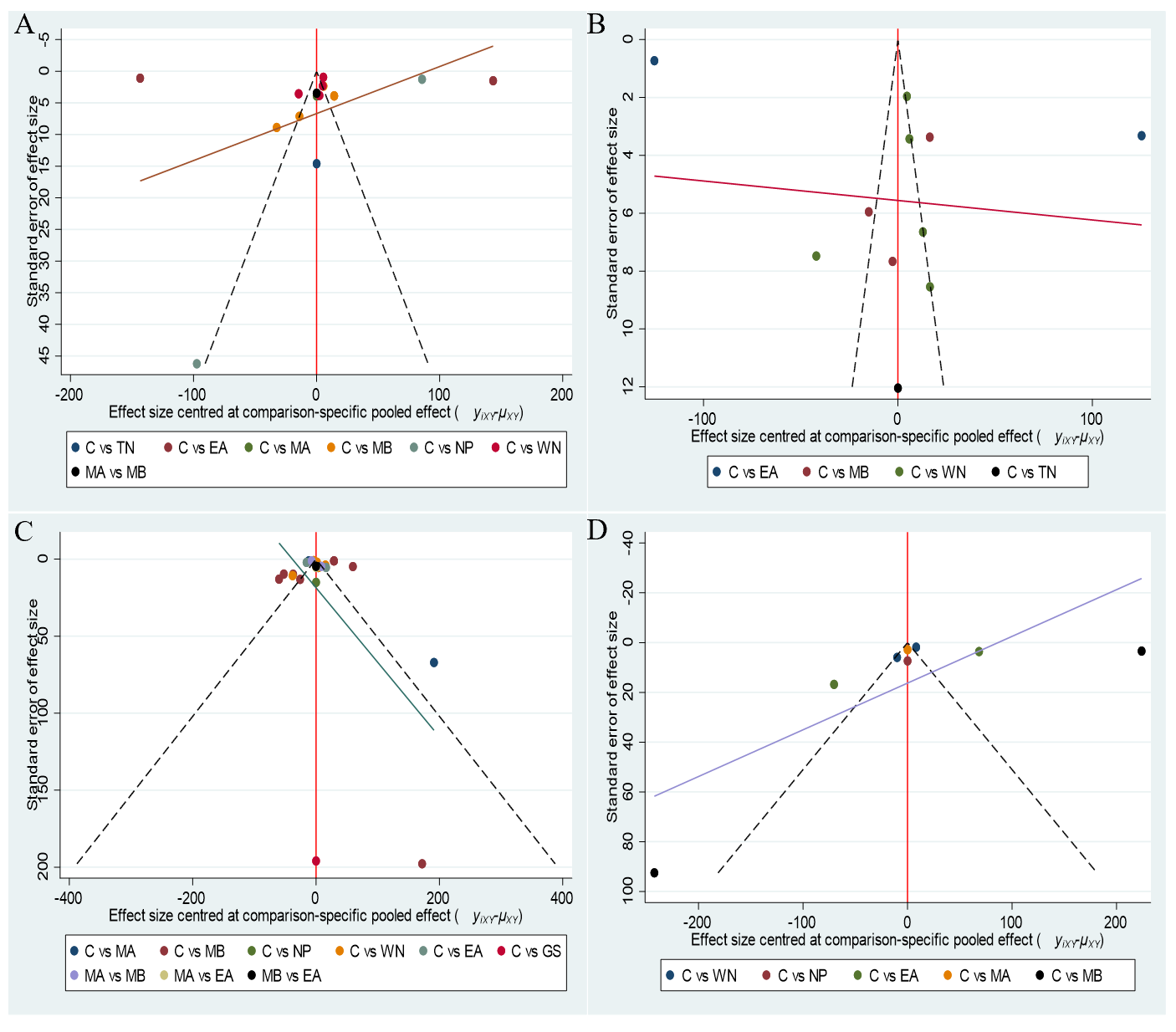
**Figure S7.3** Funnel plot for publication bias in inflammation-related outcomes. **A** IL-1βlevel; **B** IL-6 level; **C** TNF-α; **D** IFN-γ. Note: C: control group; MA: manual acupuncture; EA: electroacupuncture; MB: moxibustion; WN: warm needling therapy; NP: needle-pricking.


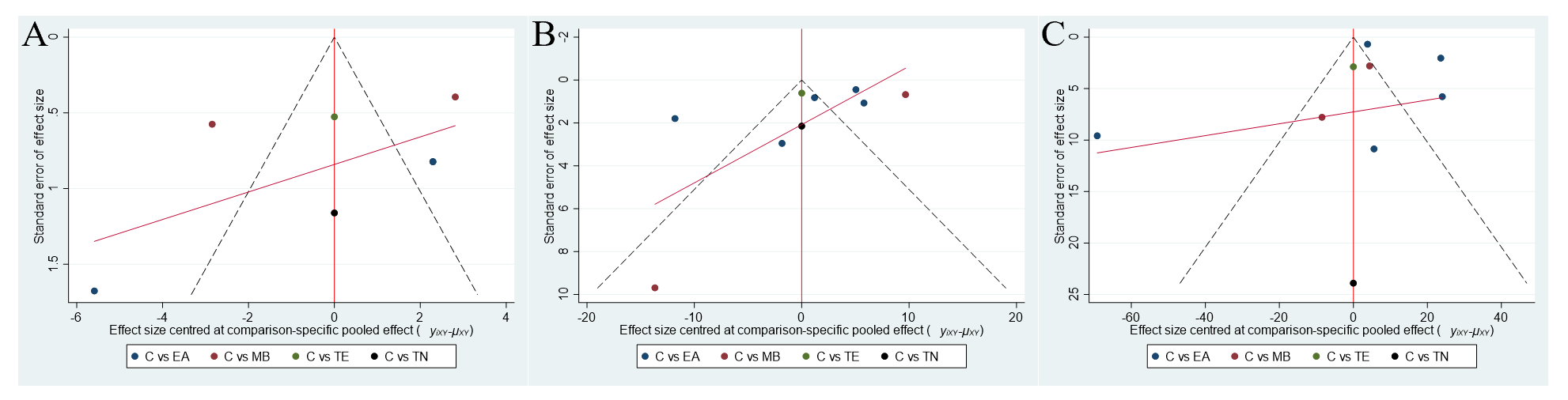
**Figure S7.4** Funnel plot for publication bias in endocrine-related outcomes. **A** CRH level; **B** ACTH level; **C** CORT level. Note: C: control group; EA: electroacupuncture; MB: moxibustion; TN: tuina; TE: thread-embedding therapy.

# **Supplementary Table S8** Summary of subgroup analysis results

| Outcomes^a^ | | Model^b^ | Intervention ＆ Mean Difference (95% CI)^c^ |
| --- | --- | --- | --- |
| Fatigue-related Outcomes | FST | Main Analysis | EA 347.00 (144.70, 549.29)  MB 311.28 (146.36, 476.20) |
|  |  | PSM + PYM | EA 255.76 (110.59, 400.93)  MB 214.41 (93.20, 335.62) |
|  | TST | Main Analysis | MA -42.31 (-75.77, -8.85)  EA -46.95 (-74.52, -19.37)  MB -44.99 (-62.08, -27.91)  GS -83.02 (-115.34, -50.70) |
|  |  | PSM + PYM | MA -43.73(-78.19, -9.27)  EA -58.79(-96.03, -21.55)  MB -49.54(-68.76, -30.32)  GS -84.73(-118.18, -51.27) |
|  | MWM | Main Analysis | MB -17.63 (-29.05, -6.22)  EA -28.42 (-39.98, -16.86)  GS -19.47 (-34.85, -4.09) |
|  |  | PSM + PYM | MB -17.61 (-29.97, -5.24)  EA -28.85 (-44.18, -13.52)  GS -19.48 (-36.14, -2.82) |
|  | OFT-V | Main Analysis | EA 9.96 (5.49, 14.42)  MA 10.51 (4.81, 16.21)  MB 7.33 (3.86, 10.81) |
|  |  | PSM + PYM | EA 6.98 (1.82, 12.13)  MA 10.89 (5.86, 15.91)  MB 8.75 (5.44, 12.05) |
|  | OFT-H | Main Analysis | EA 20.64 (9.17, 32.11)  MB 20.30 (7.31, 33.29)  MA 35.23 (14.91, 55.54) |
|  |  | PSM + PYM | EA 23.10 (5.81, 40.39)  MB 22.31 (3.94, 40.68)  MA 33.18 (5.02, 61.33) |
|  | BW | Main Analysis | TN 43.61(13.42, 73.80) |
|  |  | PSM + PYM | WE 45.75(14.71, 76.79)  EA 17.94 (0.28, 35.60)  MB 16.17 (1.90, 30.44) |
| Inflammation-related Outcomes | IL-1β | Main Analysis | TN -552.03 (-700.81, -403.25)  EA -156.59 (-259.85, -53.34) |
|  |  | PSM + PYM | EA -156.59 (-273.94, -39.24)  NP -191.32 (-380.38, -2.26) |
|  | IL-6 | Main Analysis | TN -554.77 (-693.88, -415.66)  EA -163.27 (-260.27, -66.27) |
|  |  | PSM + PYM | EA -163.20(-306.23, -20.16) |
|  | TNF-α | Main Analysis | MB -24.89(-48.40, -1.37) |
|  |  | PSM + PYM | NA |
|  | IFN-γ | Main Analysis | NA |
|  |  | PSM + PYM | NA |
| Endocrine-related Outcomes | CRH | Main Analysis | EA -9.91 (-14.77, -5.05)  TN -16.96 (-25.37, -8.55) |
|  |  | PSM + PYM | EA -15.36 (-23.81, -6.91)  TN -16.96 (-25.07, -8.85) |
|  | ACTH | Main Analysis | TN -20.02(-36.11, -3.93) |
|  |  | PSM + PYM | TN -20.02(-38.64, -1.40) |
|  | CORT | Main Analysis | EA -35.69 (-65.43, -5.95)  TN -89.95 (-170.13, -9.77) |
|  |  | PSM + PYM | EA -42.28 (-76.89, -7.68)  TN -89.95 (-172.31, -7.59) |

Note: (a) FST: exhaustion time in forced swim test; TST: immobility time in tail suspension test; MWM: escape latency in morris water maze; OFT-V: vertical score in open field test; OFT-H: horizontal score in open field test; BW: body weight. (b) PSM: physical stress model; PYM: psychological stress model. (c) MA: manual acupuncture; EA: electroacupuncture; FA: fire-acupuncture; MB: moxibustion; WN: warm needling therapy; TN: tuina; GS: guasha; TE: thread-embedding therapy; NP: needle-pricking; NA: Not applicable.
